# An 11-bp Insertion in *Zea mays fatb* Reduces the Palmitic Acid Content of Fatty Acids in Maize Grain

**DOI:** 10.1371/journal.pone.0024699

**Published:** 2011-09-13

**Authors:** Lin Li, Hui Li, Qing Li, Xiaohong Yang, Debo Zheng, Marilyn Warburton, Yuchao Chai, Pan Zhang, Yuqiu Guo, Jianbing Yan, Jiansheng Li

**Affiliations:** 1 National Maize Improvement Center of China, China Agricultural University, Beijing, China; 2 Corn Host Plant Resistance Research Unit, Agricultural Research Service, United States Department of Agriculture, Starkville, Mississippi, United States of America; 3 Genetic Resources Program, International Maize and Wheat Improvement Center, Mexico City, Mexico; Cairo University, Egypt

## Abstract

The ratio of saturated to unsaturated fatty acids in maize kernels strongly impacts human and livestock health, but is a complex trait that is difficult to select based on phenotype. Map-based cloning of quantitative trait loci (QTL) is a powerful but time-consuming method for the dissection of complex traits. Here, we combine linkage and association analyses to fine map QTL-*Pal9*, a QTL influencing levels of palmitic acid, an important class of saturated fatty acid. QTL-*Pal9* was mapped to a 90-kb region, in which we identified a candidate gene, *Zea mays fatb* (*Zmfatb*), which encodes acyl-ACP thioesterase. An 11-bp insertion in the last exon of *Zmfatb* decreases palmitic acid content and concentration, leading to an optimization of the ratio of saturated to unsaturated fatty acids while having no effect on total oil content. We used three-dimensional structure analysis to explain the functional mechanism of the ZmFATB protein and confirmed the proposed model in vitro and in vivo. We measured the genetic effect of the functional site in 15 different genetic backgrounds and found a maximum change of 4.57 mg/g palmitic acid content, which accounts for ∼20–60% of the variation in the ratio of saturated to unsaturated fatty acids. A PCR-based marker for QTL-*Pal9* was developed for marker-assisted selection of nutritionally healthier maize lines. The method presented here provides a new, efficient way to clone QTL, and the cloned palmitic acid QTL sheds lights on the genetic mechanism of oil biosynthesis and targeted maize molecular breeding.

## Introduction

The production of 817 million tons of maize in 2009 (http://faostat.fao.org) makes it one of the most important crops in the world, and it is projected to be the largest source of calories in the human diet by 2020 [1]. Maize oil production in 2004 was ∼2 million tons, a 54% increase from 1999 (http://faostat.fao.org). The proper ratio of unsaturated to saturated fatty acids in maize oil is necessary to maintain lower blood levels of serum cholesterol and low density lipoproteins, and to avoid some immunological diseases [2]. A high proportion of unsaturated fatty acids (>80% in maize kernels) may exert potential curative effects on inflammation and obesity [3]. As the major saturated fatty acid in maize grain, palmitic acid plays an important role in achieving an ideal saturated to unsaturated fatty acids ratio in corn oil.

Increasing the oil content of maize grain is an efficient alternative to increasing total maize production to boost oil supplies. High-oil maize lines are the product of long-term human selection. The first high-oil stocks, IHO (Illinois High-Oil), were created over 100 years of selection [4], [5], and an additional set of lines, BHO (Beijing High-Oil) reached the same kernel oil content after only 18 generations of a more-directed selection [6]. Understanding the underlying mechanism that led to IHO and BHO formation is very important for accelerating future high-oil maize breeding [7]. Around 50 genes or QTLs (each with small effects and mainly additive gene action) were observed underlying the IHO oil concentration [8]. In contrast, five major (and multiple minor) QTLs, with epistasis, were found to contribute to maize kernel oil concentration in BHO lines [9]. The presence of major QTLs was the main reason that the BHO population was generated so quickly, in contrast with the creation of the IHO, in which multiple minor (but no major) QTLs were found. The functional genes that make up these five major QTLs in BHO are not yet fully characterized. In fact, to date, very few functional genes underlying the variation in levels and ratios of saturated fatty acids have been reported in maize.

QTL mapping is still the most powerful tool for identifying the genomic region that controls complex quantitative traits in animals or plants. QTL fine mapping and cloning is, however, a logistical challenge. Only a few QTLs have been cloned, and these correspond to loci with major effects such as *teosinte branched1 (tb1)* [10], [11], *vegetative to generative transition 1 (vgt1)* [12], *qHO6-DGAT1 (DGAT1)* [13] and *teosinte glume architecture1 (tga1)* [14]. The publication of the complete maize genomic sequence has made association mapping based on linkage disequilibrium (LD) a more efficient method for functional gene cloning and validation [15]–[17]. There are, however, two major disadvantages in association mapping analysis: false positives caused by population structure and lower statistical power due to rare alleles present at low frequencies [18]–[20]. The combination of linkage and association analysis to identify and validate sequence variation (gene/QTL fine mapping) that is associated with beneficial phenotypes allows the exploitation of the advantages of both analyses while overcoming the limitations of each.

In a previous study, we identified the major QTL-*Pal9*, on maize chromosome 9, which accounts for 42% of the phenotypic variation of palmitic acid content in maize grain in a bi-parental segregating population [9]. The objectives of this study were to fine map QTL-*Pal9* to the level of a single gene using linkage and association analysis; to characterize the candidate gene and identify the functional variation; to verify the association in different genetic backgrounds; and to validate the functional sequence variation using *in vivo* gene expression profiling and *in vitro* complementation studies. A model explaining the underlying gene function and a PCR-based marker for marker-assisted selection of fatty acid composition in maize kernels are also presented here. These findings provide useful insights for understanding the genetic mechanism of oil biosynthesis, and targeted, efficient maize molecular breeding.

## Results

### Primary QTL mapping of *Pal9* using BC_1_S_1∶2_ and BC_2_S_2∶3_ populations

Line RIL129 was chosen from the recombinant inbred line (RIL) population derived from B73, a normal-oil inbred line, and By804, a high-oil inbred line [9]. It has the By804 allele in the target QTL and displays a high level of palmitic acid. In a BC_1_S_1∶2_ backcrossing population derived from RIL129 and B73 (recurrent parent) and segregating for the QTL-*Pal9* region, a major QTL linked to palmitic acid levels was mapped between markers LB52 and umc2213 on chromosome 9 using 129 individuals. The confidence interval around QTL-*Pal9* is 11.5 cM with a maximal logarithm of odds (LOD) score of 9.10. It explains 15%–28% of the phenotypic variation for palmitic acid content (C16∶0) and concentration (C16∶0/ALL), the ratio of saturated fatty acids (SFA/ALL), the ratio of unsaturated fatty acids (UFA/ALL) and the ratio of saturated fatty acids to unsaturated fatty acids (SFA/UFA) (Table 1). The BC_1_S_1_ population was backcrossed and selfed to create a BC_2_S_2∶3_ population with 135 individuals. Fifty-six markers scattered along the target region (Contig373; http://www.maizesequence.org/) were developed based on the predicted gene sequences (Table S1). These markers, along with 211 background markers that segregated in the parents of the original RIL population, were mapped in these 135 individuals. A QTL affecting the same traits was detected in the BC_2_S_2∶3_ population, and the genomic region containing the QTL was narrowed down to 6 cM between LB52 and LD42, which contains ∼1,400 kb. The maximum LOD score increased to 47, and *R^2^* varied from 43 to 83% for palmitic acid content and related traits (Table S2).

**Table 1:** QTL mapping results from a BC_1_S_1∶2_ population with 129 individuals.

Trait^a^	A^b^	Left Marker	Right Marker	LOD	*R^2^* (%)
C16∶0 (mg/g)	0.87	LB52	umc2213	4.05	15
C16∶0/ALL (%)	1.28	LB52	umc2213	9.10	28
SFA/ALL (%)	1.16	LB52	umc2213	4.74	16
UFA/ALL (%)	−1.18	LB52	umc2213	5.92	20
SFA/UFA (%)	2.03	LB52	umc2213	6.44	21

A major QTL between markers LB52 and umc2213 was found to be linked to all these traits using Windows QTL Cartographer with 1,000 permutations at a significance level of 0.05.

a Trait abbreviations can be found in the main text.

b The additive effect of the By804 allele.

### Development of informative recombinant lines and QTL fine mapping via combined linkage and association mapping

**Figure 1:**
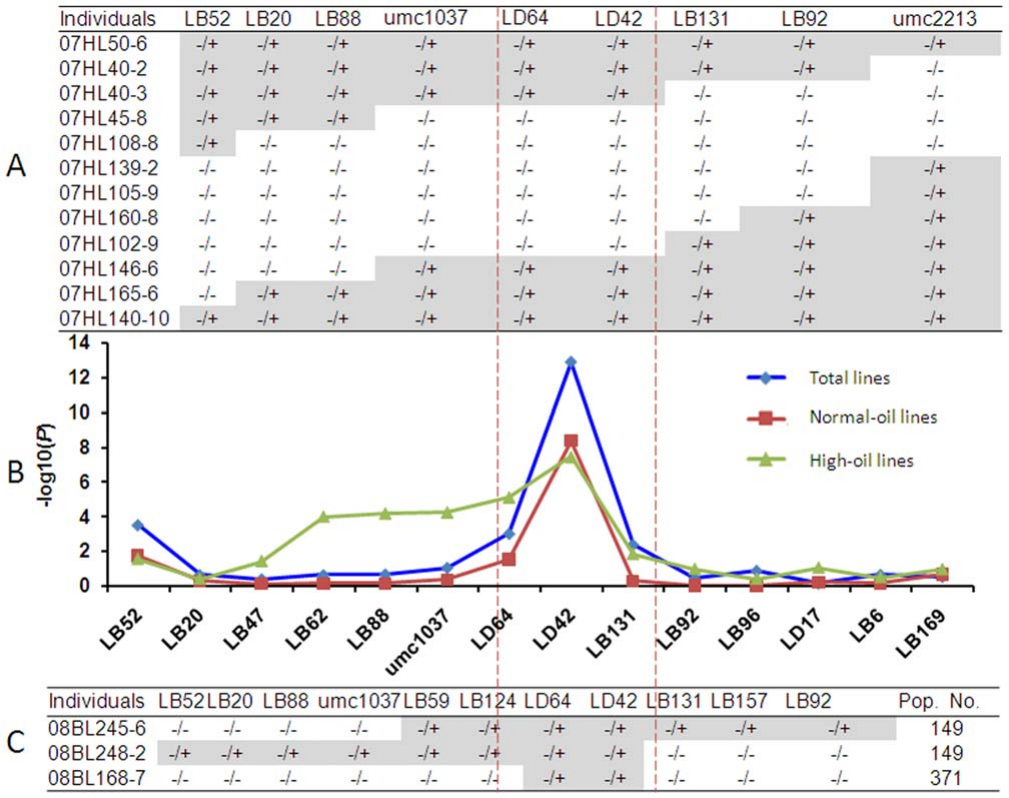
Informative recombinants identification combining association and linkage analyses. (A) Useful recombinants identified in the BC_2_S_1_ population for nine markers falling within the previously identified QTL-*Pal9*. −/− and −/+ is the homozygous allele of B73, allele that are heterozygous at the target loci, respectively. (B) Association mapping results based on 14 markers in the target genomic region for the variation of palmitic acid content in maize kernels based on association mapping in three subsets. (C) Three useful recombinants identified from two BC_3_S_1_ populations. 08BL245-6 and 08BL248-2 are from a BC_3_S_1_ population with 149 individuals of 07HL50-6. 08BL168-7 is from a BC_3_S_1_ population with 371 individuals of 07HL40-3 (lines presented in Figure 1A). −/− and −/+ is the homozygous allele of B73, allele that are heterozygous at the target loci, respectively.

A larger mapping population containing 877 lines was created in the BC_2_S_1_ generation to further fine map the region included in QTL-*Pal9*. This population was genotyped using nine markers residing in the target region between LB52 and umc2213. A number of By804 fragments overlapping introgression lines containing recombination events in the target genomic region were identified (Figure 1A).

Meanwhile, a subset of 74 elite inbred lines (including 40 normal and 34 high-oil inbred lines) was chosen from a Chinese Association Mapping Panel (CAM155) [21]. This subset contains a relatively narrow genetic background and a LD decay distance of ∼100 kb (*r^2^* ≥0.1) in the target QTL region (Figure S1). Using bioinformatics analysis, 14 PCR-based markers (Table S1) were developed based on the annotated genes within QTL-*Pal9*. All the developed markers were used to genotype the association panel and seek associations. One marker, LD42, which was developed from maize fingerprint map BAC AC218168 (http://www.maizesequence.org/), showed significant statistical association (*P* = 5.0E−14) in the panel for C16∶0 (Figure 1B). It was assumed that the underlying gene of QTL-*Pal9* is located in the region near LD42. According to the results of linkage and association analyses, two lines (07HL50-6 and 07HL40-3; Figure 1A) were selfed and backcrossed to B73 to develop BC_3_S_1_ populations. Following the screening of the two BC_3_S_1_ populations (149 individuals derived from 07HL50-6 and 371 individuals from 07HL40-3), three useful recombinants (08BL245-6, 08BL248-2 and 08BL168-7; Figure 1C) were identified and selfed to develop BC_3_S_2_ populations for progeny validation.

**Figure 2:**
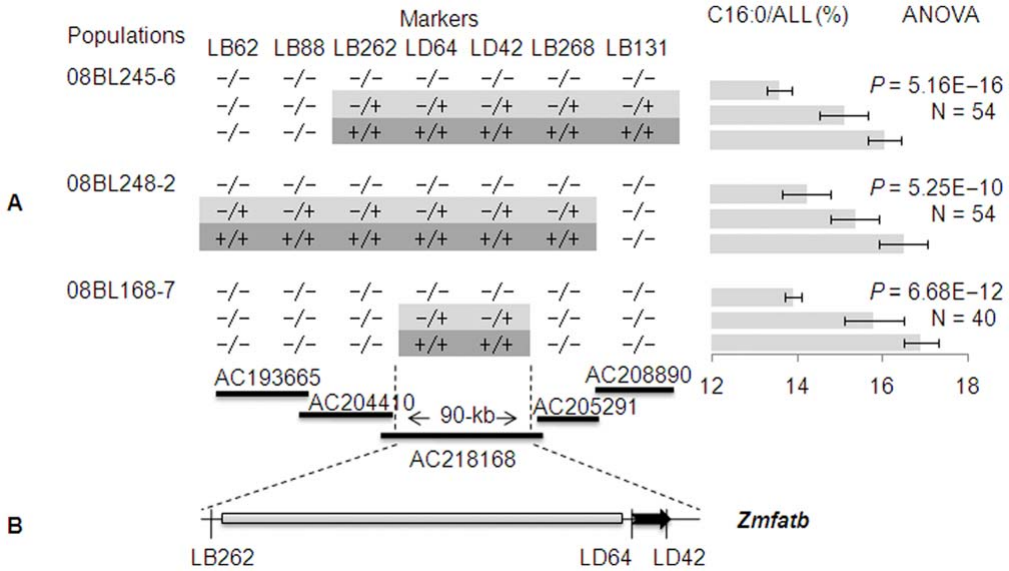
Narrowing down of QTL-*Pal9* to a 90-kb genomic region. (A) Progeny tests in BC_3_S_2∶3_ of three lines to compare the three recombinant classes that delimited QTL-*Pal9* to the region between LB262 and LB268. Progeny tests compared palmitic acid concentrations (C16∶0/ALL) using ANOVA to find significant differences between the three genotype classes. The black thicker lines represent BAC clones with their accession number in NCBI. −/−, −/+ and +/+ is the homozygous allele of B73, allele that are heterozygous for B73 and By804 and homozygous allele of By804, respectively. (B) Bioinformatics analysis of the target region, which contains only one gene, *Zmfatb* (the black arrow). The grey boxes represent repetitive sequences.

Fifty-four individuals of the BC_3_S_2∶3_ population from 08BL245-6, which contains the downstream fragment introgression from By804 in the target genomic region, were significantly different (*P* = 5.16E−16) for C16∶0/ALL among the three genotype classes (homozygous for B73, homozygous for By804 and heterozygous; Figure 2A; Table S3). The mean value of C16∶0 increased from 5.92 to 7.17 mg/g (21%), and C16∶0/ALL from 13.7 to 16.3% (19%). A large difference (*P* = 5.25E−10) for C16∶0/ALL was also observed in the BC_3_S_2∶3_ population derived from 08BL248-2 containing the upstream introgression fragments of By804 (Figure 2A; Table S3). The mean C16∶0 and C16∶0/ALL values in the individuals homozygous for the B73 allele were 5.79 mg/g and 14.5%, respectively; these values increased to 6.88 mg/g and 16.8% in the individuals homozygous for the By804 allele, an increase of 19% and 16%, respectively. A significant difference (*P* = 6.68E−12) of C16∶0/ALL was also observed in the BC_3_S_2∶3_ population derived from 08BL168-7, which contains only the 90-kb introgression from By804. Increases from 5.92 to 7.12 mg/g in C16∶0 and from 13.9 to 16.9% in C16∶0/ALL were observed between individuals homozygous for the B73 allele and individuals homozygous for the By804 allele, increases of 20% and 22%, respectively (Table S3). This locus also significantly co-segregated with the phenotypic variation of traits SFA/ALL, UFA/ALL and SFA/UFA in the above mentioned populations (Table S3). Progeny testing using BC_3_S_2∶4_ populations with larger sample sizes confirmed the significant differences between the homozygous allele classes and the heterozygous genotype (Table S4). Thus, QTL-*Pal9* was narrowed down to a 90-kb genomic region between LB262 and LB268 within AC218168 (Figure 2A).

As a final mapping step, a BC_4_S_2∶3_ population with 132 individuals derived from a BC_4_S_1_ line with only the 90-kb genomic region introgression from By804 between markers LB262 and LB268 (08BL168-7; Figure 2A) was developed for progeny testing and QTL fine mapping. QTL-*Pal9* explained 33% and 69% of the phenotypic variation for C16∶0 and C16∶0/ALL, respectively, in this population. The maximum LOD was 35 and 77% of the phenotypic variation was explained by the QTL-*Pal9* locus in these BC_4_S_2∶4_ populations (Table S5 and Figure S2). These results further indicate that the 90-kb genomic region contains the underlying gene for QTL-*Pal9*.

### *Zmfatb* is the gene underlying QTL-*Pal9*

Only one protein-coding gene was found in the target 90-kb genomic region, as all other sequences were determined to be repetitive sequences using multiple bioinformatics analysis methods (as detailed in the Materials and Methods). This gene is highly similar to *FATB* (AT1G08510) in *Arabidopsis*, which encodes the acyl-ACP thioesterase. Mutagenesis analysis of *FATB* in *Arabidopsis* has shown that the deficiency of *FATB* results in much lower palmitic acid content in leaves and leads to a smaller plant [22]. Analysis of the sequence and functional domain indicates that the candidate gene mined in this study has the same gene structure (6 exons and 5 introns) as *FATB* and a similar acyl-ACP thioesterase domain. Thus, we named the candidate gene reported here *Zmfatb (GRMZM5G829544)* and deduced that it is the gene underlying QTL-*Pal9* (Figure 2B). The markers developed within the *Zmfatb* sequence, LD42 and LD64, have the most highly significant association with the measured phenotypes (Figures 2A and 2B), which gives additional evidence that *Zmfatb* is the right candidate gene for QTL-*Pal9*.

### An 11-bp insertion/deletion is the functional site of *Zmfatb*

**Figure 3:**
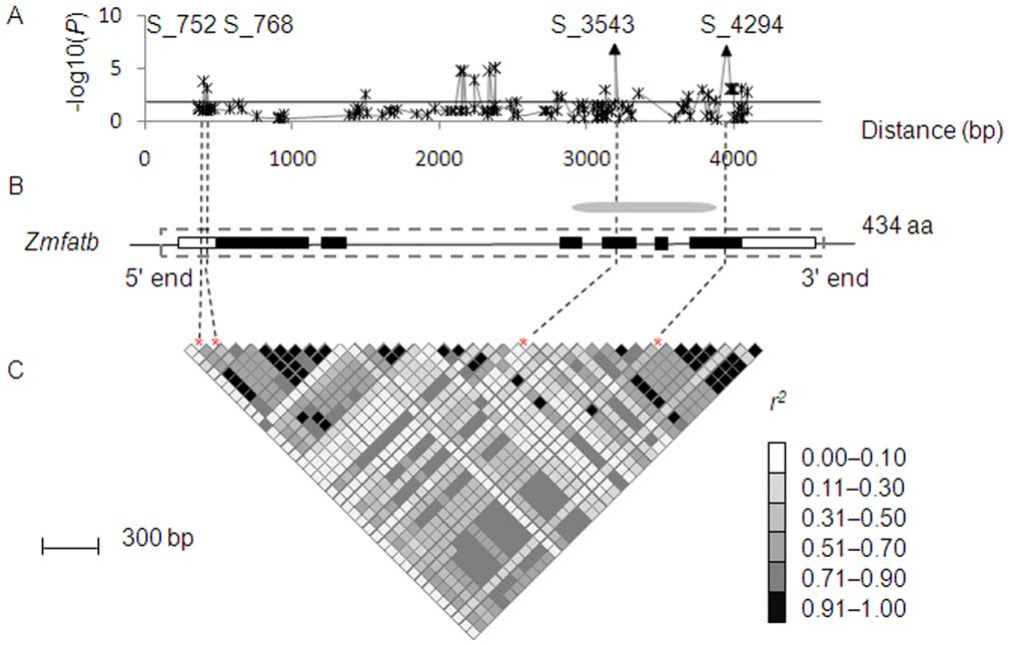
Association mapping results in CAM155 and graph of pairwise LD between significantly associated polymorphic sites of *Zmfatb*. (A) Sites that were significantly associated with variation in the palmitic acid composition in Hainan, 2007. (B) The structure and functional domain of *Zmfatb*. Filled black boxes represent exons, open boxes indicate the UTR and the gray dashed boxes mark the region sequenced in this study. And the grey ellipse represents the acyl-ACP thioesterase domain encoded by the third, fourth, fifth and sixth exons. (C) A representation of the pair-wise *r^2^* among the significantly associated sites, where darker shading of each box corresponds to a higher *r^2^*. The red stars and black dashed lines represent the most significant polymorphic loci.

The *Zmfatb* gene was sequenced in the entire association panel of 155 lines (CAM155) assembled by Yang et al. [21] (Figure 3B). In total, 16 InDels and 135 single-nucleotide polymorphisms (SNPs) were identified over a 3,796 bp sequenced gene region. Linkage disequilibrium within *Zmfatb* decayed relatively quickly (Figure S3). The average *r^2^* dropped sharply to 0.2 within only 500 bp and decayed to below 0.1 within ∼3.5 kb; this is a convenient LD decay rate for efficient association analysis of this sequence in this panel. Meanwhile, fatty acids were measured in three environments, and a broad range of variation was observed in the CAM155 panel [21], of which palmitic acid content ranged from 4.4 mg/g to 16.3 mg/g and had high heritability (96.6%).

The mixed linear model (MLM) controlling for population structure and kinship [23] was used to test associations between the detected polymorphisms and variation in five target traits (C16∶0, C16∶0/ALL, SFA/ALL, UFA/ALL and SFA/UFA) measured in three environments. Complete results from the MLM analysis can be found in Figure 3, Table 2 and Table S6. Seventeen polymorphic loci were highly associated with variation of the five target traits in CAM155 at *P*<1.0E−03 level (Figure 3A). Among the 17 significant associations, two loci (S_752 and S_768) are located in the 5′ untranslated region (UTR), one (S_4409) is located in the 3′ UTR, and all others are in introns or exons. None of the polymorphisms in the exons cause amino acid changes except S_4294, which is an 11-bp InDel in the sixth exon. S_3543 and S_4294 had the highest and most stable associations in all phenotypic datasets and are located in the same LD block (*r^2^* = 0.96; Figure 3C). The polymorphism at S_3543 occurred in an exonic region located in the functional domain but is a synonymous mutation that does not cause amino acid sequence changes. In contrast, the polymorphism at S_4294 results in an early stop codon in the protein. The S_752 and S_768 polymorphisms in the 5′ UTR are in LD with S_4294 (*r^2^* = 0.8), and had some significant associations in many phenotypic datasets (*P* = 1.0E−05). No significance was, however, detected in some environments for S_752 and S_768. Similarly, S_4409, which is located in the 3′ UTR and is in moderate LD with S_4294, shows significant association in some environments, but no significance in others. Based on these data, S_4294 in exon 6 was predicted to be the functional mutation of *Zmfatb* that causes the observed phenotypic changes in palmitic acid-related traits.

**Table 2:** Associations between palmitic acid-related traits and the 11-bp InDel of *Zmfatb*.

Panel	MAF (%)^a^	Traits^b^	*P* value	*Zmfatb* −/−	*Zmfatb* +/+	*R^2^* (%)^c^
CAM155	39^d^/82^e^	C16∶0 (mg/g)	3.09E−06	7.96±2.15	9.87±2.67	11
		C16∶0/ALL (%)	4.69E−09	13.93±2.4	17.02±2.43	25
		SFA/ALL (%)	2.17E−08	15.78±2.42	18.81±2.26	27
		UFA/ALL (%)	2.91E−08	84.12±2.44	81.11±2.26	26
		SFA/UFA (%)	5.22E−08	18.75±3.27	23.19±3.03	23
AM500	87^d^/370^e^	C16∶0 (mg/g)	1.05E−05	6.06±1.82	7.16±1.85	6
		C16∶0/ALL (%)	2.77E−09	15.02±2.00	16.9±1.74	16
		SFA/ALL (%)	4.57E−08	17.12±1.98	18.98±1.79	15
		UFA/ALL (%)	8.87E−07	81.10±2.13	79.20±1.90	5
		SFA/UFA (%)	2.45E−05	21.10± 0.03	23.96±3.21	14

a MAF, minor allele frequency.

b Trait abbreviations can be found in the main text.

c *R^2^* was calculated via ANOVA using the 2007 Hainan phenotypic data from the Chinese Association Mapping Panel, which contains 155 maize inbred lines (CAM155) [21] and 2009 phenotypic data from the Association Mapping Panel, which contains 527 maize inbred lines (AM500) [25].

d The number of inbred lines with the 11-bp insertion at the functional site.

e The number of inbred lines with the 11-bp deletion at the functional site. −/− and +/+ is the homozygous allele of B73 and homozygous allele of By804 based on the 11-bp InDel, respectively.

**Figure 4:**
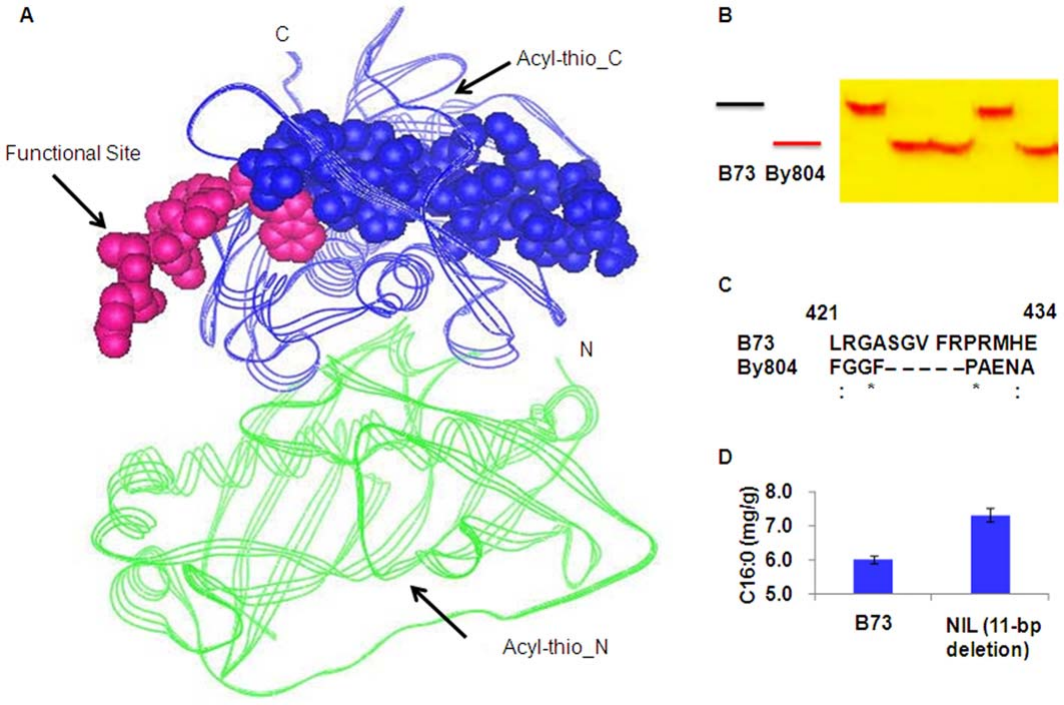
The 11-bp InDel is the functional polymorphism of *Zmfatb*. (A) Three-dimensional protein structure of ZmFATB, in which green line indicates the acyl-thioesterase N-terminal domain (Acyl-thio_N), blue line indicates the acyl-ACP thioesterase C-terminal domain (Acyl-thio_C), and the 11-bp functional site is shown in pink circles. (B) Gel image of the co-dominant marker based on the 11-bp functional site. (C) Amino acid changes in B73 and By804 caused by the 11-bp InDel. (D) Variation in palmitic acid content between the BC_4_S_3_ lines carrying the 11-bp deletion and their near-isogenic parent (B73) carrying the other haplotype of the InDel.

The B73 allele of the S_4294 InDel is 11-bp longer than the By804 allele, leading to an early stop translation in B73. Using the Swiss-Model homology modeling server and PyMOL, the three-dimensional protein structure of the ZmFATB protein was predicted based on an structural template, 2OWN, from Joint Center for Structural Genomics (not published, x-ray resolution, 2.0 Å; Figure 4A). 2OWN consists of two parts of acyl-ACP thioesterase domain in different chains A and B, which display “hot dog folds” [24]. ZmFATB shared 51% sequence identity with 2OWN (Figure S4). In maize, the catalytic domain of chain A and B span in residues 12 to 259, and Asn-17, Ser-141, Val-181 and Arg-259 together form the catalytic sites. The polymorphism at S_4294 in B73 is an 11-bp insertion (Figure 4B) that can result in an addition of 5 amino acids and a difference of 14 amino acids in the C-terminal region as compared with the By804 allele (Figure 4C and Figure S4). The different amino acids between the B73 and By804 alleles are located after residue 260 which do not reside in the acyl-ACP thioesterase domain. This may explain why the 11-bp InDel did not cause extreme phenotypic variation but only a quantitative change. The homology modeling analysis suggested that the protein fragment at the 11-bp InDel plays an important role as a portal or “door”, which controls the quantity of substrate entering into the reaction vessel “hot dog fold”. The B73 allele (increased by 5 amino acids) makes the portal smaller, which is thus less accessible to the substrate, leading to the observed decrease in palmitic acid content and variation in related traits as well (Figure 4D).

### Validation of the 11-bp InDel functional site in a larger association panel

An easy-to-use PCR-based marker for *Zmfatb* was developed from the sequence containing the 11-bp InDel (Figure 4B and Table S1) and was used to genotype a larger and more diverse association mapping panel with 527 lines (AM500) developed by Yang et al. [25]. Five hundred and two inbred lines were successfully genotyped for this polymorphism, of which 470 were homozygous and 32 were heterozygous. Only two alleles (B73 and By804) were identified with a minor allele frequency (MAF) ≥0.05, but one other allele with an even longer deletion and a frequency of 0.02 was identified. The B73 allele was present in the association mapping panel at a frequency of 0.17, and the By804 allele at 0.74. In the whole panel, the 11-bp InDel was significantly associated with the five target traits. The phenotypic variation explained by this InDel varied from 5% for UFA/ALL to 16% for C16∶0/ALL (Table 2). This provides strong additional evidence that the 11-bp InDel in the sixth exon of *Zmfatb* is the causal polymorphism of QTL-*Pal9*.

### In vivo *Zmfatb* expression analysis

Twenty days after pollination (DAP) is an important stage for fatty acid biosynthesis. Many functional lipid metabolism genes in the maize embryo are expressed at higher levels from 15 to 25 DAP; the oil percentage in the embryo increases quickly from 15 to 20 DAP in both high- and normal-oil lines [26]. As *Zmfatb* plays an important role in the early stages of fatty acid biosynthesis and because initial experiments showed some variation in *Zmfatb* expression at 20 DAP using a single individual for each near-isogenic line (NIL) genotype (Figure S5), we chose 20 DAP for gene expression profiling and further analyses.

**Figure 5:**
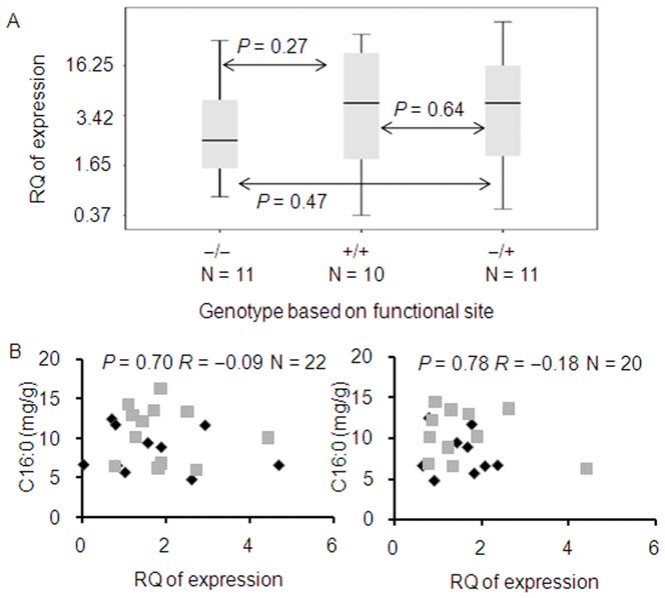
Relationship between *Zmfatb* expression level and palmitic acid content. (A) Expression analysis in BC_4_S_2∶3_ NIL with the 11-bp insertion (allelic to B73, −/−; N = 11), the 11-bp deletion (allelic to By804, +/+; N = 10) and heterozygous allele, (B73 × NIL, −/+; N = 11). RQ is the abbreviation of relative quantity. (B) Correlation analysis between *Zmfatb* expression level and palmitic acid content in diverse elite inbred lines in embryos collected at 20 days after pollination in 2008 (left) and 2009 (right). The grey boxes and black diamonds represent individuals that contain and lack the 11-bp fragment, respectively. *P* values were derived from *t*-tests; *R* values represent the correlation coefficient. RQ is the abbreviation of relative quantity.

An analysis of *Zmfatb* mRNA expression was carried out in 32 individuals in the heterozygous and both homozygous classes (B73 and By804) in the 90-kb fragment introgression NIL background (the BC_4_ generation). There were no differences in expression levels in maize kernels among the three different genotypes (Figure 5A). An additional expression test was run for a collection of over 20 inbred lines that segregate at the 11-bp InDel (Figure 5B). No difference in expression levels in maize embryos was found among the three classes of alleles over two years (*P* = 0.70, N = 22 in 2008 and *P* = 0.78, N = 20 in 2009). In addition, the correlation coefficients between the variation of *Zmfatb* expression and palmitic acid content are very low (*R* = −0.09 and −0.18 in 2008 and 2009, respectively), making it unlikely that the variation of kernel palmitic acid content is regulated by differences in transcript levels. In addition, S_752 and S_768, found in the 5′ UTR and segregating in the inbred lines, are also uncorrelated with *Zmfatb* expression variation (*P* = 0.25, N = 22 in 2008 and *P* = 0.63, N = 20 in 2009). Thus, *in vivo* expression analysis excludes the possibility that the variation seen in target phenotypes are caused by expression variation and that S_752 and S_768 are also functional sites. It does, however, give further indirect evidence that the 11-bp InDel causing the amino acid variation is the functional site.

### In vitro expression analysis of *Zmfatb* in *E. coli*

**Figure 6:**
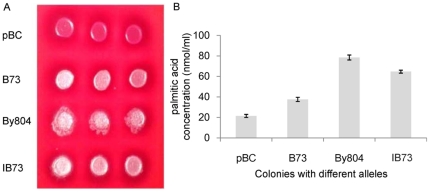
In vitro expression analysis of *Zmfatb* in *E. coli*. (A) MacConkey agar plate-based complementation screen for plant thioesterase activity using different *Zmfatb* alleles. Colonies with functional alleles show varying shades of white, whereas those containing empty vector (pBC) are pink. (B) Palmitic acid concentrations produced by different alleles of *Zmfatb* in the bacterial expression system after 36-h cultivation. Error bars represent the standard deviation for two independent clones of each allele with three replicates per clone. pBC, the empty vector; B73, *Zmfatb* B73 allele; By804, *Zmfatb* By804 allele; IB73, *Zmfatb* B73 allele containing the *in vitro*–mutagenized 11-bp deletion.

To further verify that the 11-bp InDel at S_4294 is the functional site, a range of *Zmfatb* alleles was transformed into a bacterial expression system that allows determination of enzyme activity by measuring the free fatty acid released into the medium [27]. Except for the empty vector, pBC, all *Zmfatb* alleles (B73 allele with 11-bp insertion, By804 allele with 11-bp deletion and IB73, which carries the B73 allele but with 11-bp deletion created by site-directed mutation) express active plant acyl-ACP Thioesterase (Figure 6A). Two colonies of each allele were randomly selected and tested for thioesterase activity. Preliminary experiments showed that fatty acid accumulation in the medium was significantly different between the B73 and IB73 alleles after 18 h and these differences were clearest at 36 h (Figure S6). Consequently, we picked the 36-h time point to analyze the function of the 11-bp InDel. As expected, the colonies containing the By804 allele produced the most palmitic acid; colonies containing the B73 allele produced the least palmitic acid; and IB73 had significantly higher palmitic acid than did the colonies with the unaltered B73 allele (Figure 6B). The IB73 allele improved thioesterase activity by ∼72% as compared with that of the B73 allele, and this again confirms that the 11-bp InDel is the functional polymorphism of *Zmfatb* and can affect the activity of plant acyl-ACP thioesterase.

### Validation of the *Zmfatb* effect in different genetic backgrounds

Two F_2∶3_ populations (Dan340 × K22 with 202 individuals and K22 × CI7 with 227 individuals) that segregate at the 11-bp InDel showed a significant correlation between segregation of this allele and the variation in traits C16∶0, C16∶0/ALL, SFA/ALL, UFA/ALL and SFA/UFA in maize grain. The phenotypic variation of the five target traits that was explained by the functional site varied from 34 to 51% in the Dan340 × K22 population, and 33–47% in the K22 × CI7 population (Table S7). Total oil content was, however, not changed in the maize grain according to the segregation at *Zmfatb* (*P* = 0.72 and 0.89, respectively).

**Figure 7:**
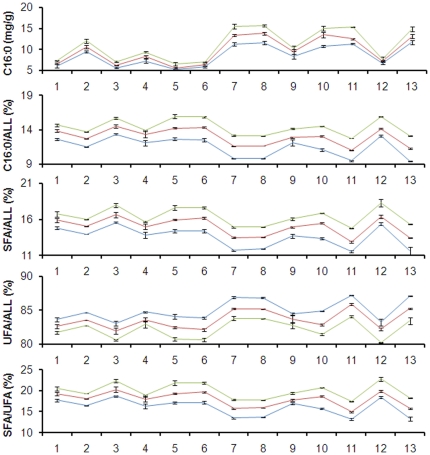
Genetic effects of the 11-bp InDel for the five palmitic acid–related traits in 13 different genetic backgrounds. The *y* axis represents the variation in levels of C16∶0, C16∶0/ALL, SFA/ALL, UFA/ALL and SFA/UFA. The different segregating populations are shown along the *x* axis (1–11, F_2_ populations; 12 and 13, BC_1_S_2_ populations): 1, 7784-4Ht × 832; 2, 7784-4Ht × Sy1035; 3, Mo17 × Ji63; 4, By4839 × Mo17; 5, 832 × Shen5003; 6, Shen5003 × U8112; 7, By813 × By804; 8, By4839 × By815; 9, 4F1 × By4839; 10, By815 × By804; 11, By804 × By815; 12, (7784-4Ht × 832) ×7784-4Ht and 13, (By804 × By815) ×By804. Blue, red and Green lines represent the homozygous allele of B73, the heterozygous allele, and the homozygous allele of By804 based on the 11-bp InDel, respectively.

Another 11 F_2_ populations and 2 BC_1_S_2_ populations were developed based on the 11-bp InDel and the pedigree information of the chosen lines in order to estimate the *Zmfatb* effect in different genetic backgrounds (Table S8). A comprehensive analysis of all populations found significant differences among the three genotypic classes of *Zmfatb* (B73 homozygous, By804 homozygous and heterozygous) for all five target traits (C16∶0, *P* = 1.77E−05; C16∶0/ALL, *P* = 6.07E−09; SFA/ALL, *P* = 1.34E−08; UFA/ALL, *P* = 9.39E−09 and SFA/UFA, *P* = 1.74E−08). Separate single-marker factor analyses of each segregating population were also used to detect significant differences for all five target traits across the three *Zmfatb* genotypes. The trends in each population were similar, although the magnitude of the effect differed in different genetic backgrounds (Figure 7 and Table S8). No QTL affecting the total oil content was detected in this region, which is consistent with the results found in the RIL (B73 × By804) and F_2∶3_ (Dan340 × K22 and K22 × CI7) populations.

## Discussion

### *Zmfatb* functional model and its utilization

The selection of *fatb* by plant breeders can be traced over the last century and *fatb* improvement is currently an active area of research. Voelker et al. first cloned the *fatb* gene in *Arabidopsis* and found that the expression of complementary DNA in seeds resulted in the accumulation of more medium chain fatty acids [28]. Topfer and Martini revealed regions of possible functional importance by comparison of deduced amino acid sequences [29], after which Salas and Ohlrogge were able to distinguish substrate specificity of FATB acyl-ACP thioesterase from FATA acyl-ACP thioesterase [30]. Use of *Arabidopsis fatb* T-DNA mutants allowed the essential role of *fatb* saturated fatty acids in plant growth to be systematically worked out [22], and *fatb* transgenic *Brassica napus* with altered fatty acid expression profiles points to the evolutionary origin of the plant acyl-ACP thioesterases [31]. To date, *fatb* genes have also been characterized in *Brassica campestris*, *Jatropha curcas* and *Diploknema (Madhuca) butyracea* [32]–[34]. However, although this trait has been well-known for many years, *fatb* genes, especially as quantitative genes, are rarely reported and to date, never knowingly used in crop improvement.

In this study, we showed that *Zmfatb* is the gene underlying the major QTL-*Pal9* that affects palmitic acid content and related traits in maize grain. *Zmfatb* is orthologous to *Arabidopsis FATB*, which encodes acyl-ACP thioesterase. It can affect the palmitic acid content of glycerolipids in multiple organisms and tissue types, including seeds [22]. The gene structure of *Zmfatb* is similar to that of *Arabidopsis FATB*, both of which contain 6 exons and 5 introns that are similar in their general organization; their protein products are also conserved, with 78.4% identity at the amino acid level. We have also shown through linkage and association analyses that an 11-bp InDel in the sixth exon of *Zmfatb* is the causal polymorphism of this QTL. The function of the 11-bp InDel was confirmed by protein structure and expression analyses, and further confirmed in different genetic backgrounds.

Although previous antisense and expression regulation studies in *Arabidopsis* and soybean demonstrated that *FATB* expression variation influences *in vivo* production and final levels of saturated fatty acids in flowers and seeds [35]–[37], we did not observe *Zmfatb* expression variation in *in vivo* expression profiling of the parents of the NIL populations or multiple inbred lines. Nevertheless, the phenotypic variation between each genotypic class of the *Zmfatb* functional site is quite large. *Zmfatb* mRNA level is very high in maize kernels (Figure S5), but the reduction of *Zmfatb* mRNA was not sufficient to reduce C16∶0 levels in certain tissues in our studies, as had been described by Bonaventure et al. [22]. Expression level differences are therefore unlikely to cause the phenotypic differences seen in this trait in maize.

The 11-bp InDel is not located in the functional domain or active sites, but the structure of the protein and the concavity at the 11-bp InDel site strongly suggest that it may play an important role as a portal or “door” that controls the quantity of substrate entering the reaction vessel. The By804 allele carries the 11-bp deletion, which corresponds to a deletion of 5 amino acids, thus making the portal bigger. This may provide greater access for the substrate (Figure 4A), leading to the observed increase in palmitic acid content and also to variability in related traits (Figure 4D). Nine elite inbred lines analyzed in the association mapping panel AM500 contained an even longer deletion; however, the palmitic acid content associated with this allele was not significantly different (*P* = 0.76) compared to lines with the By804 allele. Since the longer deletion allele is rare in this panel (MAF = 0.02), we could not estimate the genetic effect accurately, and whether or not the longer deletion further improves the accessibility for the substrate must be the target of a new study.

The deletion of 11 base pairs (and 5 amino acids) is concomitant with a change in 14 amino acids downstream of the deletion in the By804 allele. The *in vitro E. coli* complementation test further confirmed that these changes result in an increase in palmitic acid levels. The enzyme encoded by the By804 allele has a stronger enzymatic activity compared with the enzyme encoded by B73 allele (containing the 11-bp insertion, Figure S6).

As a functional gene underlying quantitative trait variation, the 11-bp InDel in the last exon of *Zmfatb* does not result in extreme phenotypic variation, possibly because the mutation did not occur in the functional domain and only affects the substrate access to the encoding enzyme (as explained above). On the other hand, there may also be other *fatb* iso-enzymes at work as well. In maize, there are two copies of *fatb* [38]; the second is located on chromosome 6 and also underlies a major palmitic acid content QTL[9]. The locus on chromosome 6 may alleviate the functional defect of QTL-*Pal9*; however, the gene effects of *Zmfatb* in the NIL population and the *E. coli* complementary test (some, but not all of which, contain a working copy of the chromosome 6 *fatb*) are almost identical. This suggests that the majority of the quantitative phenotypic variation is not caused by the complementary effect of other iso-enzymes.

Increasing the unsaturated fatty acid concentration (by decreasing the palmitic acid content) without changing the total oil content in maize grain would increase the nutritional value of the oil. Fatty acid content is currently quantified using gas chromatography, which is very expensive and inaccessible to many small laboratories and breeders from developing countries. PCR-based, user friendly markers developed from well-validated genes that underlie QTLs could be cheaply and quickly used for marker-assisted selection, even in small breeding programs [39], [40]. Here, we identified the gene and functional mutation underlying a major QTL in maize for saturated fatty acid composition that does not affect the total oil content, which will be very useful for the improvement of high-oil maize lines with a high ratio of unsaturated to saturated fatty acids. The effect of the 11-bp InDel is stable across different genetic backgrounds (Table S8) and the insertion can reduce palmitic acid levels (C16∶0) by an average of 1 mg/g, and palmitic acid concentration (C16∶0/ALL) by 20%. The 11-bp InDel at *Zmfatb* has no measured effects on other agronomic traits including total kernel oil content, kernel width and kernel length (data not shown). Thus, the user-friendly PCR-based marker developed from *Zmfatb* (Figure 4B) can be efficiently used to improve oil quality in maize breeding programs.

### Combining linkage and association mapping can speed QTL fine mapping and cloning

Although it is not difficult to map a QTL to a 5- to 10-cM interval, cloning the underlying gene from an interval this large is still a very big challenge [41]. Here we present a comprehensive protocol combining linkage and association mapping that takes advantage of current and historical recombination events for QTL cloning in those species with an available reference genome. We recommend four steps when using this strategy, as follows:

1. Map the target QTL to a small region (∼5–10 cM) using primary segregating populations (such as BC_1_, BC_2_, F_2_, F_2∶3_, etc).
2. Choose a small association mapping panel with a narrow genetic background and develop markers based on annotation information for genes in the target QTL region for association analysis. The use of 74 Chinese elite inbred lines with an LD decay of about 100 kb in the present study allowed the candidate gene region to be narrowed down to ∼100 kb. This required the use of only one or two markers per 100-kb region, and only those markers that segregate in the original QTL mapping population should be used, thus increasing the efficiency of finding the target gene. With the development of next generation sequencing techniques [42] and the associated cost decrease over traditional sequencing, two alternatives should be considered for this step: a number of appropriate fragments in the target genomic region can be sequenced in the small panel (rather than developing markers from these loci), which provides more markers per sequenced gene for the association analysis; or the entire target region can be sequenced by sequence capture technologies [43], which allows the use of all polymorphisms in the region for the association analysis.
3. Significant associations can be used to score the primary segregating populations to identify useful recombinants for validation (for example, the three overlapping recombinants in this study). Advanced backcrossed populations (BC_4_, BC_5_ or higher) are generally needed to homogenize the genetic background for QTL fine mapping and cloning; however, these populations may miss some of the informative recombinants from the early generations. In this study, one recombinant in the target genomic region was identified using only 135 individuals from the BC_2_S_2_ population, whereas 4,732 individuals from the BC_4_S_2_ population were needed to find one recombinant between the same two markers. The underlying mechanism for the variation of recombinant classes among different generations is still not very clear but may be controlled by genetic factors [44], [45].
4. Once the target QTL is mapped to a small region (in this case, 90 kb), the candidate gene may be identified via bioinformatic analysis. The function of the candidate gene(s) can then be validated using association mapping with a bigger and more diverse panel, which will also aid in the determination of the functional polymorphism(s). The decay of LD in diverse elite inbred lines in maize can be as small as 1–5 kb [46], a resolution that will quickly and efficiently bring us to the level of an individual gene. Other validation methods (expression, transformation, etc.) may be used as appropriate with the most promising gene(s).

## Materials and Methods

### Construction of the NIL population

The advanced backcross NIL population was developed as shown in Figure S7. A RIL population was constructed from a cross between B73 (low kernel oil content) and By804 (high kernel oil content) by single-seed descent [9]. RIL129 from this population, which derived 44.5% of its genetic background from B73, was the high-palmitic acid donor parent, whereas B73 was the recurrent parent. Starting from the BC_1_ population, 211 simple sequence repeat markers were used to analyze the genetic background in the backcross populations. Meanwhile, association analysis [23] using simple sequence repeat markers in the target genomic region in a natural population of 74 selected Chinese elite inbred lines was employed to select informative recombinations. The selected lines with the lowest amount of similarity to By804 in their background and carrying useful recombination events in the target genomic region were selfed for progeny validation and backcrossed to B73 to create the NIL population. Map positions used throughout this paper were based on the genetic maps created from the B73 × By804 RIL population [9] or the BC_1_S_1∶2_ populations. The details of NIL construction, genotyping and traits measurements for each generation can be found in the Method S1.

### Candidate gene mining

The gene prediction software Genscan (http://genes.mit.edu/GENSCAN.html) and Fgenesh (http://mendel.cs.rhul.ac.uk/mendel.php?topic=fgen-file) and the maize EST and plant protein databases (http://www.ncbi.nlm.nih.gov/) were used for candidate gene mining. For validation of the gene prediction, the program CENSOR (http://www.girinst.org/censor/index.php) was applied iteratively by a 10-kb increment for repetitive sequence annotation. Gene functions were predicted through Interproscan [47], KEGG [48] and Blast2GO using GO annotation [49].

### Association mapping of *Zmfatb*

The whole *Zmfatb* gene was sequenced across a panel of 155 Chinese elite inbred lines [21] (CAM155; Figure S8). All primer sequences used in this study are presented in Table S1. The alignment of all sequences for polymorphism identification was done using the multiple sequence alignment program MUSCLE [50], and was refined manually using BioEdit [51]; refined sequences were exported to Phylip [52] for further analysis. Nucleotide polymorphisms including SNPs and InDels were identified in TASSEL 2.0.1 [53] for all polymorphisms present at a frequency of ≥0.05. TASSEL 2.0.1 was also used to calculate *r^2^* among *Zmfatb* polymorphisms with 1,000 permutations, and F-tests were applied to measure the significance of LD between each pair of polymorphic loci. A co-dominant PCR marker based on the 11-bp InDel in *Zmfatb* was applied to genotype the larger panel of AM500 representing global maize diversity [25] for validation.

The CAM155 [21] were grown and evaluated at the Agronomy Farm in Beijing in the springs of 2006 and 2007 and in Hainan in the winter of 2007. The AM500 [25] was planted in Yunnan (YN, E 102°41′, N 25°01′) during the spring of 2009. Kernels from at least three mature ears with the same genotype were pooled and measured for fatty acid content according to Yang et al. [9]. The mixed linear model [23] controlling both population structure (the Q matrix) and relative kinship (the K matrix) was used to test for statistical association between phenotype and genotype in the two association panels using TASSEL 2.0.1 [53]. The genetic effects explaining the phenotypic variation were calculated through the analysis of variance between groups (ANOVA) in Excel 2007 for each locus and haplotype.

### qRT-PCR analysis of *Zmfatb* in B73, By804, NILs and the association panel

Plant tissues were collected from embryos at 20 DAP from more than 20 Chinese elite inbred lines that were planted in the springs of 2008 and 2009 in Beijing. In addition, we collected embryos at 20 DAP from 32 NILs (heterozygous or homozygous for B73 or By804 at the target locus) at the BC_4_S_2_ stage in the spring of 2009 (Beijing). Harvested tissues were frozen in liquid nitrogen and stored at −70°C until use. Total RNA was isolated using TRIzol reagent (Invitrogen) and digested with RNase-free DNase (Promega) as manufacturers' instructions. RNA was subjected to complementary DNA (cDNA) synthesis using AMV reverse transcriptase and an oligo (dT) primer (Promega). qRT-PCR for *Zmfatb* expression profiling was conducted with Ex Taq premix (Takara Shuzo). The 2^–ΔΔCT^ method [54] with three replicates was used to calculate average expression levels and standard deviations of *Zmfatb*. A maize housekeeping gene (actin) was used as an internal control. All primer sequences are listed in Table S1.

### Complementation test of *Zmfatb* in *E. coli* mutant strain K27

The coding sequences of the B73 (11-bp insertion) and By804 (11-bp deletion) *Zmfatb* alleles were amplified with primers FatBF and FatBR, and the IB73 allele, which contains the B73 allele but with the 11-bp insertion site-directed mutated to 11-bp deletion, was amplified with primers FatBF and I73R, from maize embryonic cDNA. Primer sequences are listed in Table S1. These amplified sequences were cloned into the pBC SK− phagemid (Stratagene) using the SacI and XbaI restriction sites. This resulted in a translational fusion between the N-terminal coding region of *lacZ* and coding region of *Zmfatb*.

PCR reactions contained 1 µl 10 µM of each primer, 4 µl 2.5 mM dNTPs, 1.25 U PrimeSTAR HS DNA polymerase (Takara Shuzo) and 25 µl 2 × PrimeSTAR GC buffer (Mg^2+^ plus). Thirty cycles of 98°C for 10 s, 58°C for 25 s, and 72°C for 2 min were performed. The acquired ∼1.5-kb band and pBC plasmid were cut with SacI and XbaI restriction enzymes (Fermentas Life Science) and the bands were gel purified (PureLink™ Quick Gel Extraction Kit, TIANGEN) and ligated into the pBC plasmid (Stratagene). The ligation mixtures were used to transform electrocompetent K27 cells (CGSC #5478; The Coli Genetic Stock Center at Yale). The transformation mixtures were spread on LB plates containing 30 µg/ml chloramphenicol and placed at 30°C overnight. Six colonies for each vector were sequenced to ensure that the coding sequence ligated to pBC was correct and in the same frame with the lacZ coding sequence.

To validate the difference in the thioesterase activity of different *Zmfatb* alleles, the fatty acid content in the BTNA medium (10 g/L NZ-amine and 5 g/L NaCl, pH 7.0) was measured for every *Zmfatb* allele along with accumulation over time. To ensure that the OD values of every bacterial suspension were equal at each time point, the OD values of the initial bacterial suspension were consistent, and the inoculum was added in accordance with the volume ratio of 1∶100. All time points were measured three times for two independent samples of each variant to calculate the standard deviation and repeatability of the expression (Figure S6). Two samples of each variant with the same OD value and equal inoculum volume were cultivated in conical flasks containing 600 ml of BTNA medium with 30 µg/ml chloramphenicol at 30°C for 36 h, and then the fatty acid content in the BTNA medium was measured for every *Zmfatb* allele variant. Fatty acid content of the growing medium around various cell cultures was determined by the production and measurement of fatty acid methyl esters [27]. Briefly, 22 µl of glacial acetic acid and 1 ml of 1∶1 (vol/vol) chloroform/methanol supplemented with 0.01 mg of C19∶0 (Sigma) as an internal standard were added to 0.5 ml of medium from pelleted cells corrected to give an equivalent cell density based on the OD_600_. After mixing by inversion, the phases were separated by centrifugation, and the lower phase was transferred to a fresh glass tube. The chloroform was evaporated with a stream of nitrogen, and the pellet was resuspended in 1 ml of 2% H_2_SO_4_ in methanol, after which the samples were heated to 90°C for 1 h. Samples were extracted once with 1 ml of 0.9% NaCl and 2 ml of hexane. The organic phase was transferred to a fresh tube and dried under nitrogen and then was resuspended in 400 µl of hexane. Samples (3 µl each) were analyzed on a gas chromatography for fatty acid methyl ester contact.

### Evaluation of the genetic effect of *Zmfatb* in different genetic backgrounds

The 155 elite Chinese inbred lines cluster into four major genetic groups: Beijing high-oil (BHO), Lancaster, Reid and Reid high-oil (RHO) lines [21]. From each group, two lines with the B73 allele (11-bp insertion) and two lines with the By804 allele (11-bp deletion) were chosen and crossed reciprocally. Thus, a total of sixteen elite inbred lines from both high-oil and normal-oil lines were crossed to develop segregating populations to estimate the effect of *Zmfatb* in different genetic backgrounds. Because some crosses didn't produce enough seeds, only 11 crosses from 12 unique lines were obtained (Table S9). S_1_ seeds from the 11 segregating populations were planted in the winter of 2008 in Hainan and selfed to create S_2_ ears. S_1_ plants from 2 segregating populations were backcrossed to one of their respective parents and the BC_1_S_1_ were planted in the spring of 2009 and selfed to create the BC_1_S_2_ segregating populations. Mature ears from these 11 S_2_ and 2 BC_1_S_2_ populations were harvested, and single kernels were genotyped using LD42, the PCR marker developed from the 11-bp InDel (Table S1). The classified kernels from each background were grouped according to the genotypes (B73 allele, heterozygous allele or By804 allele). Each group was randomly divided into three to six samples (depending on the number of successfully genotyped seeds) for fatty acid extraction, and each sample was measured in triplicate. A paired *t*-test comparing the B73 allele and the By804 allele in 13 segregating populations and an ANOVA for each population were used in Excel 2007 to determine the *Zmfatb* gene effect in different genetic backgrounds.

Meanwhile, two F_2∶3_ populations (derived from Dan340 × K22 and K22 × CI7) with 202 and 227 individuals, respectively, were genotyped according to the 11-bp InDel and measured for their grain lipid concentration. Single-marker factor analysis was done in the two populations for the validation of the genetic effect of *Zmfatb* on the variation of C16∶0, C16∶0/ALL, SFA/ALL, UFA/ALL and SFA/UFA.

### Prediction of three-dimensional structure for ZmFATB

We submitted the B73 ZmFATB protein sequence to the Swiss-Model homology modeling server (http://swissmodel.expasy.org/SWISS-MODEL.html) to obtain the most homologous three-dimensional model using the automatic modeling mode [55]. PyMOL (http://www.pymol.org/) was used to view the three-dimensional structure of ZmFATB.

## Supporting Information

Figure S1 **Linkage Disequilibrium decay in the genomic region of QTL-** ***Pal9*** **, with a window size  = 5 kb.** A total of 442 SNPs spaced along the target region (Contig 373, www.maizesequence.org) was employed to calculate *r^2^* in three association mapping populations, including “Total lines”, 74 randomly selected elite inbred lines; “High-oil lines”, a subset of 34 high-oil lines; and “Normal-oil lines”, a subset of 40 normal elite lines. The calculations were performed in Tassel 2.0.1 with 1,000 permutations.
(TIF)

Figure S2 **QTL mapping results in BC_4_S_2∶3_ (A) and BC_4_S_2∶4_ (B) populations containing only the 90-kb introgression between LB262 and LB268.** Trait abbreviations can be found in Table S2. Images in both populations were generated by Windows QTL Cartographer.
(TIF)

Figure S3 **LD decays in** ***Zmfatb*** **.** One hundred and fifty one polymorphic sites (SNPs and InDels) distributed along the sequence of *Zmfatb* were used to calculate *r^2^* in 155 lines of the Chinese Association Mapping Panel using TASSEL 2.0.1 with 1,000 permutations.
(TIF)

Figure S4 **Sequence alignment among 2OWN, ZmFATB in B73 and By804 and AtFATB in** ***Arabidopsis*** **.** 2OWN consisting of two chains A and B was obtained as the structural template by screening the Protein data bank. The alignment was first obtained from the MUSCLE program and then refined using ESPript. Secondary structure elements are presented on top: helices with squiggles, beta strands with arrows and turns with TT letters. Conserved residues are written in red in sequences block. Accessibility of 2OWN is rendered by a bar below: blue is accessible, cyan is intermediate, white is buried. The catalytic residues interacting with the substrate oxygen are labeled with black pentacles.
(TIF)

Figure S5 **Schematic diagram of** ***Zmfatb*** **gene expression profiling in NILs with the 90-kb introgression from By804.** Leaf, ear, 20P and 20R represent the leaf nearest to the ear, the un-pollinated ear, embryos 20 DAP and endosperms 20 DAP, respectively. All tissues were extracted from a single plant. −/−, +/− and +/+ is the homozygous allele of B73, allele that are heterozygous for B73 and By804 and homozygous allele of By804 based on the 11-bp InDel, respectively. RQ is the abbreviation of relative quantity.
(TIF)

Figure S6 **Time-courses accumulation of fatty acid for each allele of** ***Zmfatb*** **in** ***E.coli*** **system.** pBC represents the empty vector. B73, By804 are *Zmfatb* alleles of B73, By804, respectively, and IB73 represents *Zmfatb* allele of B73 containing the directly mutated 11-bp deletion.
(TIF)

Figure S7 **The schematic diagram of NIL population development for QTL-** ***Pal9*** **fine-mapping and cloning.** In this population, RIL129, a recombinant inbred line consisting of 44.5% genetic background from B73 and 55.5% from high-oil parental line By804, is the donor parent and B73 is the recurrent parent. Marker screening was applied from the BC_1_ generation and the useful recombination events in the target region were selected by association mapping and validated by progeny tests.
(TIF)

Figure S8 **Sequenced region of** ***Zmfatb*** **.** Filled black boxes represent exons, open boxes indicate the untranslated regions (UTR), and grey dashed dot box marks the region sequenced in this study. Colored arrows are the forward and reverse primers for sequencing. Primers (FatB7F/FatB7R, LD63F/LD49R, LD49F/LD51R and FatB2F/FatB2R) were overlapped for the sequencing.
(TIF)

Method S1 (PDF)

Table S1 **Primers used in this study.**
(PDF)

Table S2 **QTL mapping results in a BC_2_S_2∶3_ population.**
(PDF)

Table S3 **Phenotypic segregation of BC_3_S_2∶3_ populations derived from three introgression lines containing overlapping recombination events of QTL-** ***Pal9*** **.**
(PDF)

Table S4 **Phenotypic segregation of BC_3_S_2∶4_ populations derived from three introgression lines containing overlapping recombination events of QTL-** ***Pal9*** **.**
(PDF)

Table S5 **Phenotypic segregation in BC_4_S_2∶3_ and BC_4_S_2∶4_ populations containing the 90-kb target genomic introgression.**
(PDF)

Table S6 **Associations between palmitic acid related traits and polymorphism sites of** ***Zmfatb*** **at three environments in CAM155.**
(PDF)

Table S7 **QTL Mapping results in F_2∶3_ populations derived from Dan340 × K22 and K22 × CI7.**
(PDF)

Table S8 **Effect estimation of** ***Zmfatb*** **in different genetic backgrounds.**
(PDF)

Table S9 **Information for elite inbred lines selected for the gene effect validation of** ***Zmfatb*** **.**
(PDF)
