# Supplementary figures and images for: An 11-bp Insertion in Zea mays fatb Reduces the Palmitic Acid Content of Fatty Acids in Maize Grain
Source: PLoS One. 2011 Sep 13;6(9):e24699. doi: 10.1371/journal.pone.0024699 (PMC3172307; doi:10.1371/journal.pone.0024699)

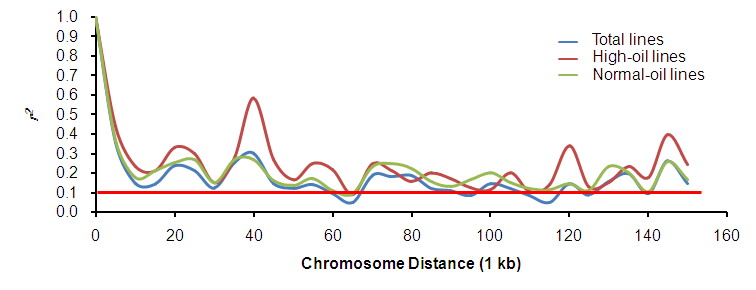

Supplement: Figure S1 — Linkage Disequilibrium decay in the genomic region of QTL- Pal9 , with a window size = 5 kb. A total of 442 SNPs spaced along the target region (Contig 373, www.maizesequence.org) was employed to calculate r2 in three association mapping populations, including “Total lines”, 74 randomly selected elite inbred lines; “High-oil lines”, a subset of 34 high-oil lines; and “Normal-oil lines”, a subset of 40 normal elite lines. The calculations were performed in Tassel 2.0.1 with 1,000 permutations. (TIF) [file pone.0024699.s001.tif]

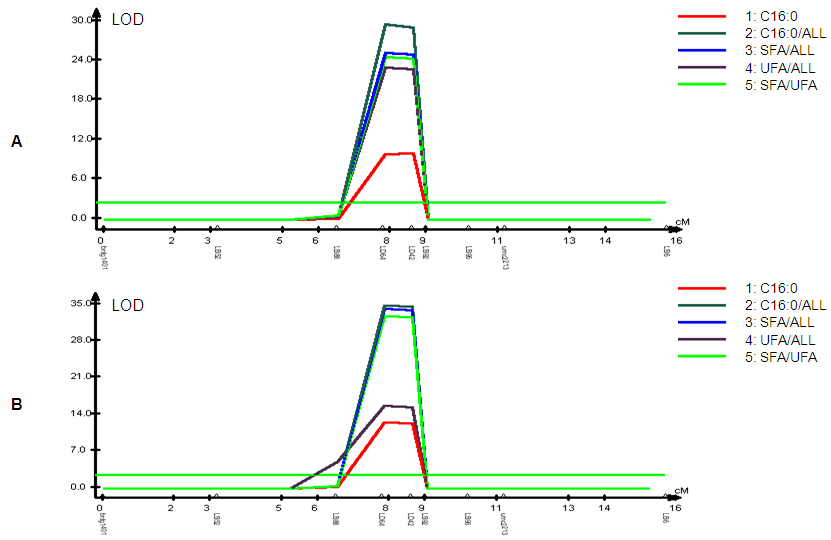

Supplement: Figure S2 — QTL mapping results in BC4S2∶3 (A) and BC4S2∶4 (B) populations containing only the 90-kb introgression between LB262 and LB268. Trait abbreviations can be found in Table S2. Images in both populations were generated by Windows QTL Cartographer. (TIF) [file pone.0024699.s002.tif]

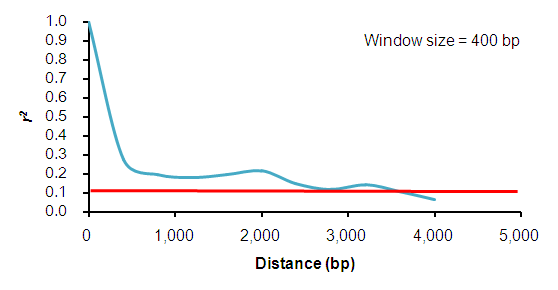

Supplement: Figure S3 — LD decays in Zmfatb . One hundred and fifty one polymorphic sites (SNPs and InDels) distributed along the sequence of Zmfatb were used to calculate r2 in 155 lines of the Chinese Association Mapping Panel using TASSEL 2.0.1 with 1,000 permutations. (TIF) [file pone.0024699.s003.tif]

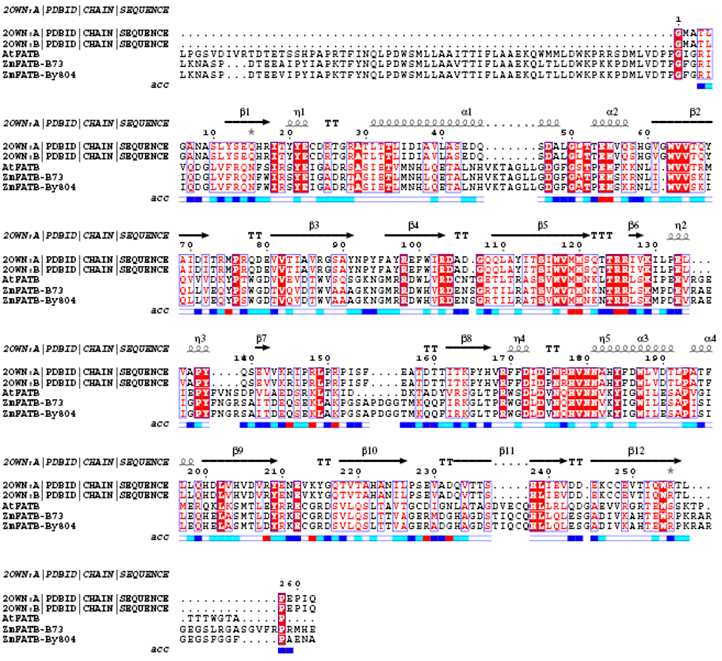

Supplement: Figure S4 — Sequence alignment among 2OWN, ZmFATB in B73 and By804 and AtFATB in Arabidopsis . 2OWN consisting of two chains A and B was obtained as the structural template by screening the Protein data bank. The alignment was first obtained from the MUSCLE program and then refined using ESPript. Secondary structure elements are presented on top: helices with squiggles, beta strands with arrows and turns with TT letters. Conserved residues are written in red in sequences block. Accessibility of 2OWN is rendered by a bar below: blue is accessible, cyan is intermediate, white is buried. The catalytic residues interacting with the substrate oxygen are labeled with black pentacles. (TIF) [file pone.0024699.s004.tif]

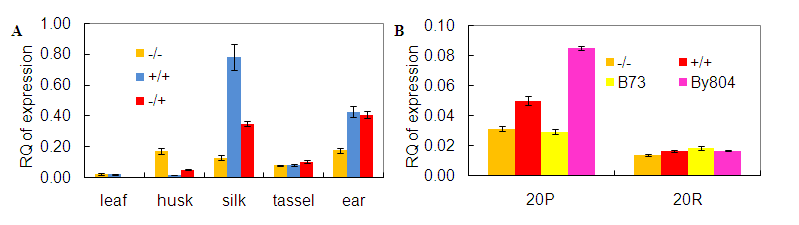

Supplement: Figure S5 — Schematic diagram of Zmfatb gene expression profiling in NILs with the 90-kb introgression from By804. Leaf, ear, 20P and 20R represent the leaf nearest to the ear, the un-pollinated ear, embryos 20 DAP and endosperms 20 DAP, respectively. All tissues were extracted from a single plant. −/−, +/− and +/+ is the homozygous allele of B73, allele that are heterozygous for B73 and By804 and homozygous allele of By804 based on the 11-bp InDel, respectively. RQ is the abbreviation of relative quantity. (TIF) [file pone.0024699.s005.tif]

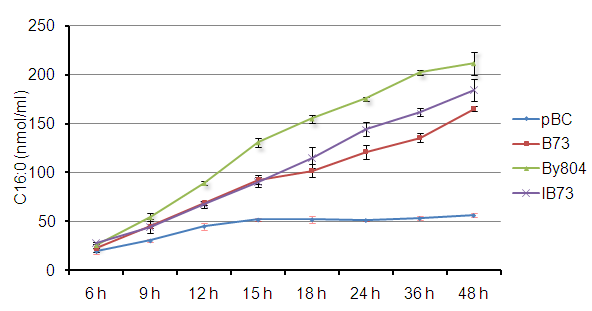

Supplement: Figure S6 — Time-courses accumulation of fatty acid for each allele of Zmfatb in E.coli system. pBC represents the empty vector. B73, By804 are Zmfatb alleles of B73, By804, respectively, and IB73 represents Zmfatb allele of B73 containing the directly mutated 11-bp deletion. (TIF) [file pone.0024699.s006.tif]

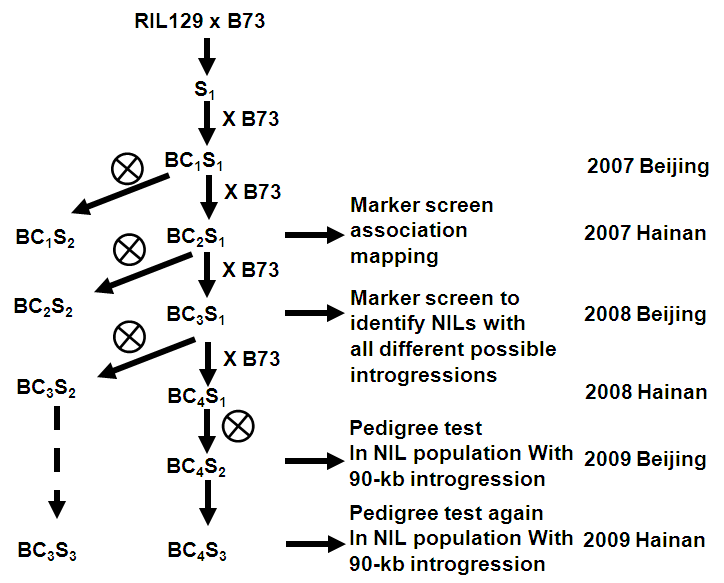

Supplement: Figure S7 — The schematic diagram of NIL population development for QTL- Pal9 fine-mapping and cloning. In this population, RIL129, a recombinant inbred line consisting of 44.5% genetic background from B73 and 55.5% from high-oil parental line By804, is the donor parent and B73 is the recurrent parent. Marker screening was applied from the BC1 generation and the useful recombination events in the target region were selected by association mapping and validated by progeny tests. (TIF) [file pone.0024699.s007.tif]

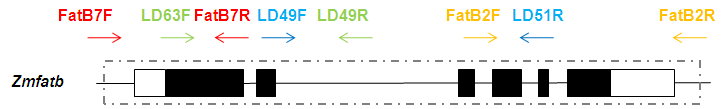

Supplement: Figure S8 — Sequenced region of Zmfatb . Filled black boxes represent exons, open boxes indicate the untranslated regions (UTR), and grey dashed dot box marks the region sequenced in this study. Colored arrows are the forward and reverse primers for sequencing. Primers (FatB7F/FatB7R, LD63F/LD49R, LD49F/LD51R and FatB2F/FatB2R) were overlapped for the sequencing. (TIF) [file pone.0024699.s008.tif]
