# Supplementary material for: An 11-bp Insertion in Zea mays fatb Reduces the Palmitic Acid Content of Fatty Acids in Maize Grain
Source: PLoS One. 2011 Sep 13;6(9):e24699. doi: 10.1371/journal.pone.0024699 (PMC3172307; doi:10.1371/journal.pone.0024699)
Supplement: Method S1 — (PDF) [file pone.0024699.s009.pdf]

# Method S1

## Construction and phenotyping of the NIL population

In the spring of 2007, $BC_1S_1$ populations were planted at the Agronomy Farm of the China Agricultural University in Beijing (Beijing, BJ, E 116°46', N 39°92') to obtain 267 $BC_1S_1$ individuals to screen the target genomic region. According to the genotype in the target region, 129 individuals were chosen to self-cross or to backcross to B73. We harvested 129 mature ears and measured each individual for palmitic acid content as well as other fatty acids in the kernels. The genetic variation of fatty acid content and composition in a population of $BC_1S_{1:2}$ plants were mapped to a genetic map using Composite Interval Mapping (Zeng, 1994) with 1,000 permutations at a significance level of 0.05 using Windows QTL Cartographer (http://statgen.ncsu.edu/qtlcart/WQTLCart.htm). Meanwhile, $BC_1$ individuals carrying the target genotype in the genomic region of interest (depending on the QTL mapping results) were screened with 211 background markers to identify individuals with the most number of markers akin to B73 for the next backcross selection.

In the winter of 2007, $BC_2S_1$ populations were planted at the Agronomy Farm of the China Agricultural University in Hainan (Hainan, HN, E 108°56', N 18°09'). Eight hundred and seventy-seven $BC_2S_1$ individuals were genotyped to identify lines containing recombinations in the target genomic region. Meanwhile, 74 inbred lines from the Chinese elite association mapping population were genotyped using high-density SNP markers in the target genomic region, and an association analysis was run on the variation of the maize kernel palmitic acid content and composition. According to the association analysis results, individuals with informative recombination in the target genomic region (i.e., recombination around the marker showing the highest significant association with palmitic acid content) were identified; these were backcrossed with B73 and self-crossed. Individuals with high genetic background recovery (markers of the same genotype as B73) that were still heterozygous at the target region were selected to produce the next generation.

In the spring of 2008, $BC_3S_1$ and $BC_2S_2$ populations derived from the chosen $BC_2S_1$ individuals were planted in Beijing. The $BC_3S_1$ populations containing the entire By804 target genomic region (according to genotypic data of the $BC_2S_1$ parents) were genotyped to identify lines with overlapping recombination events in the target loci with high association to the palmitic acid-related traits. Meanwhile, MAS was used to select individuals with a high B73 background recovery for further backcrossing and self-crossing. The $BC_3S_1$ populations with informative recombinations in the target genomic region (according to the genotype of the parents) were genotyped again to seek further recombinations. The individuals with the smallest By804 genome introgression and highest palmitic acid content and composition were identified to be backcrossed and self-crossed. At the same time, the $BC_2S_2$ populations from the parent with the highest background recovery (B73) were genotyped and phenotyped for QTL fine-mapping.

In the winter of 2008 in Hainan, $BC_4S_1$ and $BC_3S_2$ populations containing two overlapping introgressions and one single introgression were selected. Overlapping introgressions were defined for adjacent target markers. For example, if adjacent markers are called A, B, and C, overlapping introgression would be A-B and B-C; in addition, only B introgressions from By804 were evaluated. The $BC_4S_1$ population whose parent contained the smallest By804 introgression segment was genotyped, and the population with background markers that were all identical to those of B73 was chosen for further backcrossing and self-crossing. The $BC_3S_2$ populations containing the three overlapping introgressions (representing the genomic region upstream of the target introgression, downstream of the introgression and only the target locus introgression) were all self-crossed, genotyped and phenotyped on an individual-plant basis. Progeny tests were performed to narrow down the functional gene for the variation of maize kernel palmitic acid content and composition.

In the spring of 2009 in Beijing, a $BC_4S_2$ population containing the smallest By804 fragment (about 90-kb) in the target locus was planted to confirm the effect of this introgression.

In the winter of 2009 in Hainan, the $BC_3S_3$ families containing the overlapping

introgression segments and the $BC_4S_3$ families from the smallest target introgression were planted for further progeny test validation.

**Field experiments, genotyping and trait measurements**

$BC_xS_1$ and $BC_xS_2$ populations from each backcrossed and self-crossed generation were planted in 3-m-long rows with 0.6 m between rows. A randomized complete-block design was used for $BC_xS_3$ family populations in two environments with one replicate each, in which each genotype was planted in a plot with 11 plants in a row. All populations were planted at the Agronomy Farms of the China Agricultural University in the springs of 2007, 2008 and 2009 in Beijing and in the winters of 2007, 2008 and 2009 in Hainan in series. Normal agronomic practices were applied in field management.

Fresh leaves from each individual in each $BC_xS_1$ and $BC_xS_2$ population and leaves from 11 plants that were bulked together from each $BC_xS_3$ population were collected. DNA extraction was performed using the cetyltrimethylammonium bromide (CTAB) method (Rogers et al., 1985). A DNA concentration of 25 ng/μl was used for genotyping and PCR products were separated and visualized via polyacrylamide gel electrophoresis and agarose gel electrophoresis. Target genomic sequences were obtained from the B73 genomic sequence (www.maizesequence.org). SSRHunter (Li and Wang, 2005) was used to scan loci for SSR sequences, and Primer3 (Rozen et al., 2000) and Primer5 (www.Premier5BioSoft.com) were used to design primers to amplify the SSRs. Polymorphisms were identified between B73 and By804 to identify informative recombination events. Information on the primers can be found in Supplementary Table 1.

Kernels in the middle part of the mature ear from $BC_xS_1$ and $BC_xS_2$ individuals were harvested, and bulked kernels from at least four mature ears with the same genotype in each $S_3$ population were prepared for measurement of kernel fatty acid content. Two sub-samples were measured at the same time by HP6890 or HP7890 gas chromatography (Agilent Technologies). Method described by Sukhija and Palmquist (1988) was used to extract lipids with some modifications (Yang et al., 2010a). The fatty acid composition was determined according to Yang et al. (2010a). Although this

method can detect eleven fatty acids components content (Supplementary Figure 9), palmitic acid, stearic acid, oleic acid, linoleic acid and linolenic acid which account for more that 99.7% of total oil in maize kernel (Yang et al., 2010a), were selected for the QTL fine-mapping and further data analysis.

## Supplementary references

Li Q, and Wang JM (2005) SSRHunter：Development of a local searching software for SSR sites. Hereditas 27(5): 808–810.

Rogers SO, and Bendich AJ (1985) Extraction of DNA from milligram amounts of fresh, herbarium and mummified plant tissues. Plant Mol Biol 5:69–76.

Rozen S, and Skaletsky H (2000) Primer3 on the WWW for general users and for biologist programmers. Methods Mol Biol 132: 365–386.

Sukhija PS, and Palmquist DL (1988) Rapid method for determination of total fatty acid content and composition of feedstuffs and feces. J Agric Food Chem 36: 1202–1206.

Zeng ZB (1994) Precision mapping of quantitative trait loci. Genetics 136(4): 1457–1468.
