# Supplementary material for: An 11-bp Insertion in Zea mays fatb Reduces the Palmitic Acid Content of Fatty Acids in Maize Grain
Source: PLoS One. 2011 Sep 13;6(9):e24699. doi: 10.1371/journal.pone.0024699 (PMC3172307; doi:10.1371/journal.pone.0024699)
Supplement: Table S1 — Primers used in this study. (PDF) [file pone.0024699.s010.pdf]

Table S1. Primers used in this study.

Name	BAC	Genome Position	Gene	Gene Position	Function Description	Forward	Reverse
Li29	AC195188	19854800	GRMZM2G014240	3'	vaculor H+-pyrophosphatase	GGACAAGTGCGACTACGGC	GCTGCTGGGATATGGGAGA
Li31	AC195188	19854800	GRMZM2G047138	4th exon	hypothetical protein	TTCGCTCGGTTTCTTTTGC	GCTGCTCTGCTGCCTCTGA
LB25	AC195993	20682900		intergenic		AACGCGGCTCGTGTTATCT	CGTCATCTGCCCGCTTGG
LB27	AC195993	20682900	GRMZM2G056256	1st intron	hypothetical protein	GGTGTCGGAGCTGGTATTGG	ATACACGGCTCATTTGGTCG
LB74	AC194303	21006300		intergenic		AATCTTCAGCATCCATGGGG	CAGTTCAAGCCATCGGTCA
LB77	AC195890	21520800		intergenic		CATCCATCGTCGATCAAACC	AAGAGTTGGTCGTCCATGTGG
LB68	AC190756	21662900	GRMZM2G398642	8th intron	beta-carotene hydroxylase	GCCGTTCGTTCGTCTTTCG	TGCTGATTGGCGTATTTGGAT
LB70	AC190756	21662900		intergenic		TTGCCAGCGACCGAGAA	CTACAGCCACGACGAACAGG
LB78	AC195882	21746200	GRMZM2G104760	2nd intron	BRASSINOSTEROID INSENSITIVE 1-associated receptor kinase	GTCCAATAGCTGCCACCATG	GGATATGTTGTCATTGGGAAGTC
LB67	AC192170	22525300	GRMZM2G131842	5'	thiol protease SEN102	TAAGCGTTCTCACCCACCAG	AAAACAATAGCGTACCTTCCATG
LB144	AC209785	22971200	AC211152.4_FGT007	5'	hypothetical protein	CCTTCACGTGGAGAGAGACC	GACACCGTCCATTTGGTCAT
LB44	AC198662	23348500		intergenic		GAGACTGCGGCTGATGCG	GCCCAGACCTCGTTTTCG
LB148	AC206266	23544500	GRMZM2G703580	3'	hypothetical protein	AGTTTTGTCAAATTTTCGAGGA	AAAGACGCGAGAATGAATGC
LB150	AC210245	23632700	GRMZM2G122793	5'	ferredoxin-thioredoxin reductase catalytic chain	AGGATATTGCGCCATAAATAGTA	TTGTTGGAGTTTTATGTTTGTGC
bnlg244	AC198696	23799300		intergenic		GATGCTACTACTGGTCTAGTCCAGA	CTCCTCCACTCATCAGCCTTGA
LB152	AC207360	24117800	AC207360.3_FG001	5'	hypothetical protein	GAATGAATGGACGACGAGAAGA	GCGTGCGATGGAAGAAGC

LB154	AC211180	24519600	GRMZM2G102521	8th exon	solute carrier family 35 member C2	AGCTAGACGACATGCACTTCC	TTTGCTGTTTGGAAATTGGA
bnlg1401	AC218010	24157000		intergenic		CACTCGGTTTTTGCTTAGCC	GTGTCGTCGAGTGCATGC
LB156	AC208905	24686200	GRMZM2G028594	1st intron	hypothetical protein	GCCGCCTATTCACTTCGC	CTCGTCTCCGCTGCCTCT
LB52	AC201977	24828300	GRMZM2G103179	2nd intron	hypothetical protein	TGAAGGAAGCAATAATCAGAGCG	TGTGAAGCGGCGAGAAGG
LB20	AC190913	24960600	GRMZM2G005522	3'	transferase	ATGTTGCCATTGTAACGGTGAA	GGAGTTCGGGACGGTGGAG
LB62	AC190913	24960600	GRMZM2G005461	1st exon	ribosomal protein L40	AAACCAAATTCGTCCACGGG	AGCCTTGGCGACTCGGG
LB47	AC199959	25524100	GRMZM2G077040	3'	hypothetical protein	ACCTTACGCATTCGGTAGAGC	CGGTTATCATCGAGAAAATCCAG
LB59	AC199959	25524100	GRMZM2G050971	1st intron	iron-sulfur assembly protein IscA	AAAACCCACCAAAAGAACGAT	GTCTCCACAAGCCAAAACCA
LB88	AC193665	25965100	GRMZM2G106683	3'	zinc carboxy peptidase	CGTAGGAAAACGATACAACACCC	TTAATAACAACAAATATGCTGCC
umc1037	AC193665	25965100		intergenic		CTTCTTCGTAAAGGCATTTTGTGC	GTGCGGGATTCCTTAGTTTGC
LB124	AC204410	26161100	GRMZM2G173615	5'	hypothetical protein	ACAAGGCAGGCAGGGTCA	TCTTCTGGCAGTGCTCGTAGTA
LB296	AC204410	26161100	GRMZM2G173615	1st exon	POAL Cysteine-rich peptide	GCAGAGGTCGGTGAAACATC	TGGCAGTGCTCGTAGTAGGA
LB262	AC204410	26161100	GRMZM2G000645	11th intron	coatomer complex subunit	ATCGGCACTGGCAAATAAAA	GGCCACAGATGGAGAAAGAT
LD64	AC218168	26234600	FatB	5'	Acyl-ACP thioesterase	TTGCCGCCCTCCAAAACT	GGCTTCCAGAACCGTGATA
LB266	AC218168	26234600	FatB	2nd intron	Acyl-ACP thioesterase	TCAGCCATAACAGACGAGCA	GCATTTGGCAACAATCACGA
LB267	AC218168	26234600	FatB	4th intron	Acyl-ACP thioesterase	GCCCGAATCCCTTTCATTTA	CACGACGTTGTAAAACGACCGCGTG GCCGTCCAT
LD42	AC218168	26234600	FatB	6th exon	Acyl-ACP thioesterase	CGGGGCCGACATAGTGAA	CAAACTTCTGCGTTAGCATACC
LB268	AC218168	26234600	FatB	3' downstream intergenic	Acyl-ACP thioesterase	TCGAATCGCACACCTCCTG	GCAAAGCAAGCCAACCG
LB131	AC205291	26376700	GRMZM2G404897	3'	zinc finger protein	TCCCACTTTTTAGGCCAGA	CGATCACCATGGTCCCTACT

LB97	AC197529	26646200	GRMZM2G377199	5'	lectin protein kinase, putative, expressed	CAACCGCAGAGTACAAGTTACAGGG	CCTTGTACGTCGTTGCGCC
LB157	AC210692	26950000	GRMZM2G018849	3'	hypothetical protein	AAATTGGATGGATGGGAAATC	ACAGCCTAAACAACAACAGAAGTG
LB92	AC189054	27454700	GRMZM2G447455	5'	Kinesin-II 85 kDa subunit, putative	CTCGCACGTCGCACCTCCT	CGTGCAGCCGCTAGGATCG
LB96	AC193668	27582100	GRMZM2G449056	3'	transcription factor Myb2	CGTTGTTTCTGCCTGATTTCC	GGCGACCTGGACTACTGGCT
LB95	AC193668	27582100	AC193668.3_FG006	7th intron	hypothetical protein	ACAGCCATGCTTCAC	GCTGCCATTACTGATA
umc2213	AC214663	27650700		intergenic		TGATTTGATCCATTACCATCCTATTC	TGCGTGTGTCTGTCTGTCTGTTAT
LB158	AC210041	27876100	AC210041.3_FG002	2nd intron	aminomethyltransferase	CTCCTAGCAGTGAGTTCCGTGAT	AGACAGACTGATGGTCATTTTCG
LB159	AC211662	27993700	GRMZM2G398341	15th intron	phosphatidylinositol kinase	TCTTTACAGGGATTGGGTCA	CTAGTCGCGGCTGACCTCT
LB18	AC190908	28753200	GRMZM2G404603	5'	hypothetical protein	ACCCCTCTGCCGATTTCC	TCCTGCCTCGGCGTTG
LD17	AC190908	28753200	GRMZM2G024993	3rd intron	granule-bound starch synthase precursor	ACAAGGACGCCTGGGACA	GGAACAGTGGGTGGTCAA
LB194	AC204379	28954100	GRMZM2G171395	2nd intron	NAM protein	GGCCACGAGGAACACGA	CCGGCCATTTCCAACAAC
LB6	AC189782	29125600	GRMZM2G121333	3rd exon	valacyclovir hydrolase	GCACCGCATTGTATTTGGAT	TGAGCGTGGAGGCATAGGAG
LB188	AC211766	29365700	GRMZM2G118687	5th exon	peptidyl-prolyl cis-trans isomerase-like	TTTTCGGTTGATGATTTGGT	GCTGCTTGGCGTAGGTG
LB46	AC192201	29968400	GRMZM2G467992	1st intron	plasma membrane H+-ATPase subunit H	AAGTTGGAGGATGATTGC	AGGATGTAAAGGGGAAGA
LB177	AC207551	30821000	GRMZM2G071602	5th exon	protein binding protein	GCCCAGGTGGTGTAAAGGT	CATCAAAACGAAGAGGACGAG
LB196	AC202398	31203200	GRMZM2G383540	3rd intron	DAG protein	AACGGCATACGAAACAGCAT	GGTCTAGCAAATTGGATGGGT
LB198	AC189077	31551100	GRMZM2G083716	3rd intron	CPN60B	AGTGCTGGCGGTTCCC	GGCCTGCAAAACTTGTATGA
LB169	AC210074	32060700	GRMZM2G093291	5'	pentatricopeptide (PPR)	GGAAGGGAAGCGAGATAAGG	GATCCCCTCACTTGTGGCTA

					repeat-containing protein-like		
LB166	AC210296	32658500		intergenic		GAGATTCCTGTGCCAGCTTC	GAGCGGAGAGGTACCAACAG
LB183	AC195601	33878600	GRMZM2G153792	10th exon	acetyl-CoA synthetase	CGAAAGCCGTCCCACC	CACTCACGACCTGTTGTCTCCT
LB171	AC206582	34084400	GRMZM2G052389	9th exon	acyl-CoA oxidase	ATTCCTGAAGCTCCATTCCA	AAGCCCACTGCTGTTCAAGT
**RT-PCR primers**							
LD48	AC218168	26234600	FatB	RT-PCR	Acyl-ACP thioesterase	CGATACCCTACATTGCTCCC	CGAATCCAGAAGTTTTGCCTA
**Primers used in prokaryotic expression**							
FatBF	AC218168	26234600	FatB	Prokaryotic expression	Acyl-ACP thioesterase	GAGCTC GAAGCACGGTCGAGCCATGGCTGGC	
FatBR	AC218168	26234600	FatB	Prokaryotic expression	Acyl-ACP thioesterase	TCTAGA ATTGCCTCCAAGCCTCCC	
I73	AC218168	26234600	FatB	Prokaryotic expression	Acyl-ACP thioesterase	TCTAGA GGTCATTCATGCATTCTCGG	
**Sequence Primers**							
LD49F/ LD51R	AC218168	26234600	FatB	Sequencing	Acyl-ACP thioesterase	ACTTCTGGATTCGGTCCTATG	AAGATTGTGCGTCCAGAGTTT
LD63F/ LD49R	AC218168	26234600	FatB	Sequencing	Acyl-ACP thioesterase	TCTAGAGGCTTCTTTGAATCCCACA	TCAAGTTTCGTTTGCTCATCT
FatB7	AC218168	26234600	FatB	Sequencing	Acyl-ACP thioesterase	ACGCGTTTCTGTTCATAGCC	GCCAAGTTCTTTGATGTTTTGG
FatB2	AC218168	26234600	FatB	Sequencing	Acyl-ACP thioesterase	GGGAGATACTGTTCAAGTGGAC	CATGCATGATGCTCAGCTCTA
