# Supplementary material for: An 11-bp Insertion in Zea mays fatb Reduces the Palmitic Acid Content of Fatty Acids in Maize Grain
Source: PLoS One. 2011 Sep 13;6(9):e24699. doi: 10.1371/journal.pone.0024699 (PMC3172307; doi:10.1371/journal.pone.0024699)
Supplement: Table S2 — QTL mapping results in a BC2S2∶3 population. (PDF) [file pone.0024699.s011.pdf]

Table S2. QTL mapping results in a $BC_2S_{2:3}$ population.

Trait	A	D	Left Marker	Right Marker	LOD	R^2 (%)
C16:0 (mg/g)	0.58	0.004	LB52	LD42	16.56	43
C16:0/ALL (%)	1.58	–0.102	LB52	LD42	46.56	83
SFA/ALL (%)	1.47	–0.114	LB52	LD42	39.10	77
UFA/ALL (%)	–1.51	0.158	LB52	LD42	32.10	72
SFA/UFA (%)	1.97	–0.186	LB52	LD42	38.01	78

Results delimit the QTL-*Pal9* to a narrow genomic region between LB52 and LD42. These were calculated with Windows QTL Cartographer with 1,000 permutations at the 0.05 significance level. "A" represents the additive effect of By804 allele and "D" is the dominant effect. LOD represents Logarithm of odds. C16:0, palmitic acid content; C16:0/ALL, palmitic acid concentration; SFA/ALL, the ratio of saturated fatty acids; UFA/ALL, the ratio of unsaturated fatty acids; SFA/UFA, the ratio of saturated fatty acids to unsaturated fatty acids.
