# Supplementary material for: An 11-bp Insertion in Zea mays fatb Reduces the Palmitic Acid Content of Fatty Acids in Maize Grain
Source: PLoS One. 2011 Sep 13;6(9):e24699. doi: 10.1371/journal.pone.0024699 (PMC3172307; doi:10.1371/journal.pone.0024699)
Supplement: Table S3 — Phenotypic segregation of BC3S2∶3 populations derived from three introgression lines containing overlapping recombination events of QTL- Pal9 . (PDF) [file pone.0024699.s012.pdf]

Table S3. Phenotypic segregation of $BC_3S_{2:3}$ populations derived from three introgression lines containing overlapping recombination events of QTL-*Pal9*.

Population	Traits	*Pal9* –/–	*Pal9* +/–	*Pal9* +/+	A	D	R^2 (%)	*P* value
08BL245-6	C16:0 (mg/g)	5.92 ± 0.17	6.68 ± 0.31	7.17 ± 0.20	0.56	0.07	74.1	1.08E–15
	C16:0/ALL (%)	13.70 ± 0.30	15.34 ± 0.60	16.29 ± 0.40	1.12	0.17	74.9	5.16E–16
	SFA/ALL (%)	15.98 ± 0.19	17.55 ± 0.57	18.44 ± 0.36	1.06	0.17	78.9	5.70E–18
	UFA/ALL (%)	84.02 ± 0.19	82.45 ± 0.57	81.56 ± 0.36	–1.06	–0.17	78.9	5.70E–18
	SFA/UFA (%)	19.02 ± 0.17	21.28 ± 0.23	22.61 ± 0.12	1.92	0.28	78.2	1.41E–17
08BL248-2	C16:0 (mg/g)	5.79 ± 0.31	6.34 ± 0.26	6.88 ± 0.12	0.54	0.00	63.4	7.57E–12
	C16:0/ALL (%)	14.45 ± 0.60	15.59 ± 0.60	16.81 ± 0.60	1.20	–0.02	56.7	5.25E–10
	SFA/ALL (%)	16.73 ± 0.65	17.71 ± 0.65	18.66 ± 0.44	0.95	0.01	48.2	5.26E–08
	UFA/ALL (%)	83.27 ± 0.65	82.29 ± 0.65	81.34 ± 0.44	–0.95	–0.01	48.2	5.26E–08
	SFA/UFA (%)	20.09 ± 0.64	21.52 ± 0.63	22.94 ± 0.72	1.03	0.01	48.0	5.65E–08
08BL168-7	C16:0 (mg/g)	5.92 ± 0.12	6.58 ± 0.29	7.12 ± 0.47	0.57	0.03	66.8	1.38E–09
	C16:0/ALL (%)	13.89 ± 0.20	15.78 ± 0.71	16.87 ± 0.40	1.29	0.20	75.1	6.68E–12
	SFA/ALL (%)	15.97 ± 0.31	17.88 ± 0.93	19.04 ± 0.55	1.35	0.19	66.8	1.40E–09
	UFA/ALL (%)	84.03 ± 0.31	82.12 ± 0.93	80.96 ± 0.55	–1.35	–0.19	66.8	1.40E–09
	SFA/UFA (%)	19.01± 0.27	21.77 ± 0.95	23.52 ± 0.68	2.01	0.26	66.2	1.88E–09

Fifty-four, fifty-four and forty individuals were genotyped and phenotyped for target traits, respectively for a $BC_3S_{2:3}$ population derived from 08BL245-6 covering the downstream introgression from By804, a $BC_3S_{2:3}$ population derived from 08BL248-2 covering the upstream genome introgression and a $BC_3S_{2:3}$ population containing only the 90-kb introgression. ANOVA test in Excel was used to detect significance for the phenotypic segregations and gene effects of the homologous alleles of B73, By804 and the heterozygous alleles of QTL-*Pal9*. “A” represents the additive effect of By804 allele and “D” is the dominant effect. –/–, +/– and +/+ is the homozygous allele of B73, allele that are heterozygous for B73 and By804 and homozygous allele of By804 based on target genomic markers, respectively. The abbreviations of traits can be found in Table S2.
