# Supplementary material for: An 11-bp Insertion in Zea mays fatb Reduces the Palmitic Acid Content of Fatty Acids in Maize Grain
Source: PLoS One. 2011 Sep 13;6(9):e24699. doi: 10.1371/journal.pone.0024699 (PMC3172307; doi:10.1371/journal.pone.0024699)
Supplement: Table S5 — Phenotypic segregation in BC4S2∶3 and BC4S2∶4 populations containing the 90-kb target genomic introgression. (PDF) [file pone.0024699.s014.pdf]

Table S5. Phenotypic segregation in $BC_4S_{2:3}$ and $BC_4S_{2:4}$ populations containing the 90-kb target genomic introgression.

Population	Traits	*Pal9* –/–	*Pal9* +/–	*Pal9* +/+	A	D	R^2	*P* value
$BC_4S_{2:3}$	C16:0 (mg/g)	5.37 ± 0.52	6.19 ± 0.69	6.52 ± 0.48	0.53	0.14	32.5	2.81E–10
	C16:0/ALL (%)	13.86 ± 0.51	15.34 ± 0.78	17.06 ± 0.79	1.66	–0.06	69.3	1.85E–29
	SFA/ALL (%)	16.21 ± 0.56	17.72 ± 0.85	19.33 ± 0.93	1.59	–0.03	63.6	2.52E–25
	UFA/ALL (%)	82.15 ± 0.63	80.65 ± 0.92	78.94 ± 1.06	–1.66	0.05	60.3	3.51E–23
	SFA/UFA (%)	19.73 ± 0.14	21.97 ± 0.13	24.49 ± 1.01	2.03	–0.06	62.6	1.24E–24
$BC_4S_{2:4}$	C16:0 (mg/g)	6.00 ± 0.62	6.70 ± 0.59	7.36 ± 0.54	0.61	0.02	41.3	7.08E–13
	C16:0/ALL (%)	13.52 ± 0.52	14.61 ± 0.61	16.27 ± 0.52	1.53	–0.17	77.4	1.35E–34
	SFA/ALL (%)	15.65 ± 0.56	16.83 ± 0.55	18.36 ± 0.54	1.45	–0.11	76.7	6.11E–34
	UFA/ALL (%)	82.76 ± 0.83	81.58 ± 1.23	80.17 ± 0.58	–1.35	0.07	48.8	5.21E–16
	SFA/UFA (%)	18.91 ± 0.23	20.63 ± 0.16	22.91 ± 0.04	1.93	–0.15	75.2	1.53E–32

One hundred and thirty two BC_4S_2 individuals were analyzed via ANOVA test in Excel combining with the genotypic segregation of QTL-*Pal9* and the phenotypic variation of C16:0, C16:0/ALL, SFA/ALL, UFA/ALL and SFA/UFA. One hundred and twenty one pooled kernels with the same genotype in a $BC_4S_{2:4}$ family from 1,043 $BC_4S_{2:3}$ populations were measured and analyzed for the phenotypic segregation according to the QTL-*Pal9* alleles. "A" represents the additive effect of By804 allele and "D" is the dominant effect. –/–, +/– and +/+ is the homozygous allele of B73, allele that are heterozygous for B73 and By804 and homozygous allele of By804 based on target genomic markers, respectively. The abbreviations of traits can be found in Table S2.
