# Supplementary material for: An 11-bp Insertion in Zea mays fatb Reduces the Palmitic Acid Content of Fatty Acids in Maize Grain
Source: PLoS One. 2011 Sep 13;6(9):e24699. doi: 10.1371/journal.pone.0024699 (PMC3172307; doi:10.1371/journal.pone.0024699)
Supplement: Table S6 — Associations between palmitic acid related traits and polymorphism sites of Zmfatb at three environments in CAM155. (PDF) [file pone.0024699.s015.pdf]

Table S6. Associations between palmitic acid related traits and polymorphism sites of *Zmfatb* at three environments in CAM155.

Site	Features		Environments	2006 Beijing		2007 Beijing		2007 Hainan		R^2 (%)	*P* Value
			Traits	ALL[a]	EHOL[b]	ALL	EHOL	ALL	EHOL	2007HN	2007HN
S_752	Location	promoter	C16:0	1.50E–04	n.s.	1.30E–03	n.s.	1.43E–04	n.s.	11	3.00E–04
	Genotype	C/G	C16:0/ALL	4.03E–05	7.49E–04	1.30E–03	7.56E–04	1.38E–04	3.90E–03	13	6.60E–05
	Frequency(%)	36/64	SFA/ALL	2.64E–04	2.80E–03	5.60E–03	3.87E–04	1.28E–04	2.20E–03	15	1.38E–05
	AA Change	no	UFA/ALL	5.19E–04	4.60E–03	5.60E–03	3.87E–04	1.51E–04	2.30E–03	15	1.66E–05
			SFA/UFA	5.43E–04	3.60E–03	7.90E–03	4.59E–04	2.58E–04	2.60E–03	13	1.08E–04
S_768	Location	promoter	C16:0	4.23E–04	n.s.	5.00E–03	n.s.	6.73E–04	n.s.	15	7.54E–05
	Genotype	2/4/0	C16:0/ALL	1.68E–04	2.40E–03	5.60E–03	3.20E–03	4.44E–04	2.00E–03	22	7.73E–07
	Frequency(%)	40/35/25	SFA/ALL	1.30E–03	1.01E–02	n.s.	2.30E–03	4.11E–04	7.74E–04	24	2.58E–07
	AA Change	no	UFA/ALL	2.20E–03	1.63E–02	n.s.	2.30E–03	5.08E–04	9.34E–04	23	3.49E–07
			SFA/UFA	2.20E–03	1.63E–02	n.s.	2.30E–03	5.08E–04	9.34E–04	23	3.49E–07
S_4294	Location	exon6	C16:0	2.50E–06	1.20E–05	3.14E–05	3.53E–05	3.09E–06	5.55E–05	11	1.11E–03
	Genotype	0/11	C16:0/ALL	2.09E–09	1.07E–07	1.10E–08	8.71E–09	4.69E–09	1.66E–07	25	1.47E–07
	Frequency(%)	32/68	SFA/ALL	2.94E–07	1.01E–05	6.87E–07	3.72E–08	2.17E–08	1.30E–06	27	6.47E–08
	AA Change	yes	UFA/ALL	7.96E–06	7.78E–05	6.87E–07	3.72E–08	2.91E–08	1.62E–06	26	8.00E–08
			SFA/UFA	7.96E–06	7.78E–05	6.87E–07	3.72E–08	5.22E–08	1.62E–06	23	8.00E–08

[a] includes 155 elite inbred lines in the Chinese Association Mapping Panel (CAM155). [b] excludes the 34 high-oil lines from the Chinese Association Mapping Panel (CAM155). The abbreviations of traits can be found in Table S2.
