# Supplementary material for: An 11-bp Insertion in Zea mays fatb Reduces the Palmitic Acid Content of Fatty Acids in Maize Grain
Source: PLoS One. 2011 Sep 13;6(9):e24699. doi: 10.1371/journal.pone.0024699 (PMC3172307; doi:10.1371/journal.pone.0024699)
Supplement: Table S7 — QTL Mapping results in F2∶3 populations derived from Dan340 × K22 and K22 × CI7. (PDF) [file pone.0024699.s016.pdf]

Table S7. QTL Mapping results in $F_{2:3}$ populations derived from Dan340 × K22 and K22 × CI7.

Populations	N	Traits	*Zmfatb* –/–	*Zmfatb* +/–	*Zmfatb* +/+	A	D	R^2 (%)	*P* value
Dan340 × K22	202	C16:0 (mg/g)	6.628 ± 0.774	7.274 ± 0.895	7.847 ± 0.960	0.609	0.037	33.56	2.21E–09
		C16:0/ALL (%)	14.221 ±1.351	15.645 ±1.274	17.032 ±1.365	1.378	–0.021	51.38	1.94E–15
		SFA/ALL (%)	17.101 ±1.317	18.423 ± 1.245	19.726 ± 1.332	1.325	–0.011	49.35	1.20E–14
		UFA/ALL (%)	82.982 ±1.312	81.568 ±1.252	80.296 ± 1.331	–1.296	0.010	49.35	1.20E–14
		SFA/UFA (%)	20.623 ±1.948	22.544 ±1.879	24.631 ±2.026	1.931	–0.051	49.27	1.28E–14
		Oil (mg/g)	46.874 ± 4.931	46.887 ± 5.416	46.458 ± 6.099	n.d.	n.d.	n.d.	0.72
K22 × CI7	227	C16:0 (mg/g)	6.366 ± 0.806	7.050 ± 0.776	7.407 ± 0.670	0.520	0.164	33.03	2.02E–08
		C16:0/ALL (%)	14.013 ±1.182	14.521 ±1.179	16.361 ± 1.225	1.226	–0.613	46.86	1.84E–12
		SFA/ALL (%)	17.561 ±1.282	17.991 ±1.015	19.652 ±1.217	1.124	–0.618	44.35	1.17E–11
		UFA/ALL (%)	82.526 ±1.213	82.118 ±1.010	80.412 ±1.227	–1.123	0.617	44.35	1.17E–11
		SFA/UFA (%)	21.212 ±1.789	21.828 ±1.586	24.420 ±1.992	1.681	–0.941	43.85	1.68E–11
		Oil (mg/g)	45.842 ± 7.058	48.825 ± 6.700	45.654 ± 5.021	n.d.	n.d.	n.d.	0.89

"A" represents the additive effect of By804 allele and "D" is the dominant effect. P value was calculated according to the variation of the 11-bp InDel. –/–, +/– and +/+ is the homozygous allele of B73, allele that are heterozygous for B73 and By804 and homozygous allele of By804 based on the 11-bp InDel, respectively. The abbreviations of traits can be found in Table S2. n.d.is the abbreviation of not detected.
