# Supplementary material for: An 11-bp Insertion in Zea mays fatb Reduces the Palmitic Acid Content of Fatty Acids in Maize Grain
Source: PLoS One. 2011 Sep 13;6(9):e24699. doi: 10.1371/journal.pone.0024699 (PMC3172307; doi:10.1371/journal.pone.0024699)
Supplement: Table S8 — Effect estimation of Zmfatb in different genetic backgrounds. (PDF) [file pone.0024699.s017.pdf]

Table S8. Effect estimation of *Zmfatb* in different genetic backgrounds.

Populations	N	Observed N	Traits	*Zmfatb* –/–	*Zmfatb* +/–	*Zmfatb* +/+	*P* value
Mo17 × Ji63	83	18	C16:0 (mg/g)	5.649 ± 0.003	6.213 ± 0.002	7.103 ± 0.002	1.46E–10
			C16:0/ALL (%)	13.358 ± 0.003	14.496 ± 0.069	15.715 ± 0.030	3.32E–12
			SFA/ALL (%)	15.563 ± 0.011	16.637 ± 0.159	18.000 ± 0.085	1.45E–09
			UFA/ALL (%)	83.067 ± 0.007	81.967 ± 0.176	80.620 ± 0.100	2.92E–09
			SFA/UFA (%)	18.731 ± 0.022	20.297 ± 0.127	22.313 ± 0.042	1.82E–09
By4839 × Mo17	69	17	C16:0 (mg/g)	7.215 ± 0.118	8.259 ± 0.257	9.354 ± 0.003	2.84E–02
			C16:0/ALL (%)	12.065 ± 0.145	13.283 ± 0.184	13.888 ± 0.006	1.53E–06
			SFA/ALL (%)	13.858 ± 0.159	15.006 ± 0.164	15.656 ± 0.011	1.88E–06
			UFA/ALL (%)	84.758 ± 0.261	83.608 ± 0.262	82.964 ± 0.013	2.87E–05
			SFA/UFA (%)	16.421 ± 0.111	17.987 ± 0.135	18.923 ± 0.041	3.10E–06
832 × Shen5003	268	13	C16:0 (mg/g)	5.181 ± 0.002	5.499 ± 0.006	6.572 ± 0.027	4.71E–04
			C16:0/ALL (%)	12.625 ± 0.045	14.224 ± .007	15.948 ± 0.072	1.76E–09
			SFA/ALL (%)	14.421 ± 0.044	15.944 ± 0.013	17.658 ± 0.122	1.50E–08
			UFA/ALL (%)	84.042 ± 0.049	82.492 ± 0.025	80.732 ± 0.131	2.23E–08
			SFA/UFA (%)	17.159 ± 0.038	19.328 ± 0.010	21.927 ± 0.127	1.95E–08
Shen5003 × U8112	277	15	C16:0 (mg/g)	5.754 ± 0.008	6.296 ± 0.010	6.965 ± 0.001	2.25E–05
			C16:0/ALL (%)	12.369 ± 0.079	14.365 ± 0.019	15.877 ± 0.011	7.46E–12
			SFA/ALL (%)	14.323 ± 0.065	16.197 ± 0.009	17.736 ± 0.022	4.68E–12
			UFA/ALL (%)	83.991 ± 0.085	82.151 ± 0.051	80.546 ± 0.016	6.32E–11
			SFA/UFA (%)	17.121 ± 0.036	19.687 ± 0.017	22.019 ± 0.021	5.02E–12
By813 × By804	241	15	C16:0 (mg/g)	11.095 ± 0.002	13.275 ± 0.011	15.668 ± 0.017	1.48E–10
			C16:0/ALL (%)	9.777 ± 0.003	11.626 ± 0.001	13.082 ± 0.001	2.52E–19
			SFA/ALL (%)	11.682 ± 0.008	13.545 ± 0.002	14.927 ± 0.001	2.09E–17

			UFA/ALL (%)	86.899 ± 0.167	85.184 ± 0.002	83.815 ± 0.005	5.16E−10
			SFA/UFA (%)	13.413 ± 0.011	15.861 ± 0.012	17.811 ± 0.003	2.26E−16
By4839 × By815	87	15	C16:0 (mg/g)	11.569 ± 0.016	13.845 ± 0.011	15.687 ± 0.009	1.13E−09
			C16:0/ALL (%)	9.829 ± 0.001	11.646 ± 0.000	13.071 ± 0.002	3.27E−20
			SFA/ALL (%)	11.916 ± 0.004	13.582 ± 0.001	14.956 ± 0.003	1.11E−17
			UFA/ALL (%)	86.823 ± 0.003	85.152 ± 0.003	83.779 ± 0.009	2.54E−16
			SFA/UFA (%)	13.672 ± 0.008	16.013 ± 0.007	17.883 ± 0.001	1.60E−17
4F1 × By4839	245	10	C16:0 (mg/g)	8.100 ± 0.349	9.406 ± 0.051	9.816 ± 0.006	1.75E−02
			C16:0/ALL (%)	11.646 ± 0.053	12.922 ± 0.008	14.221 ± 0.009	2.38E−07
			SFA/ALL (%)	13.787 ± 0.210	14.995 ± 0.016	16.059 ± 0.032	4.74E−05
			UFA/ALL (%)	85.348 ± 0.046	83.365 ± 0.858	82.773 ± 0.026	4.92E−03
			SFA/UFA (%)	16.411 ± 0.012	18.017 ± 0.082	19.413 ± 0.011	7.92E−04
By815 × By804	78	9	C16:0 (mg/g)	10.766 ± 0.005	13.493 ± 0.048	15.030 ± 0.027	1.57E−04
			C16:0/ALL (%)	11.070 ± 0.027	13.024 ± 0.010	14.484 ± 0.005	1.02E−07
			SFA/ALL (%)	13.373 ± 0.024	15.467 ± 0.012	16.876 ± 0.000	6.00E−08
			UFA/ALL (%)	84.911 ± 0.015	82.871 ± 0.023	81.454 ± 0.003	8.87E−08
			SFA/UFA (%)	15.697 ± 0.012	18.681 ± 0.032	20.722 ± 0.068	5.40E−08
By804 × By815	87	9	C16:0 (mg/g)	11.279 ± 0.003	12.494 ± 0.004	15.287 ± 0.001	2.02E−07
			C16:0/ALL (%)	9.498 ± 0.008	10.986 ± 0.003	12.760 ± 0.002	4.14E−09
			SFA/ALL (%)	11.548 ± 0.024	12.903 ± 0.012	14.745 ± 0.003	1.26E−07
			UFA/ALL (%)	87.227 ± 0.035	85.891 ± 0.027	84.099 ± 0.009	8.33E−07
			SFA/UFA (%)	13.219 ± 0.092	15.015 ± 0.072	17.524 ± 0.038	1.43E−07
7784-4Ht × 832	92	9	C16:0 (mg/g)	6.056 ± 0.417	6.592 ± 0.190	7.302 ± 0.206	5.59E−03
			C16:0/ALL (%)	12.629 ± 0.129	13.839 ± 0.181	14.673 ± 0.168	1.36E−05
			SFA/ALL (%)	14.832 ± 0.183	15.886 ± 0.277	16.733 ± 0.337	4.39E−04
			UFA/ALL (%)	83.649 ± 0.233	82.616 ± 0.290	81.714 ± 0.286	3.83E−04

			SFA/UFA	17.675 ± 0.308	19.213 ± 0.411	20.492 ± 0.519	4.39E−04
7784-4Ht × Sy1035	89	9	C16:0 (mg/g)	9.487 ± 0.191	10.655 ± 0.365	12.008 ± 0.481	4.65E−04
			C16:0/ALL (%)	11.538 ± 0.024	12.688 ± 0.071	13.671 ± 0.038	8.76E−09
			SFA/ALL (%)	13.980 ± 0.025	15.080 ± 0.063	15.999 ± 0.059	1.80E−08
			UFA/ALL (%)	84.661 ± 0.018	83.547 ± 0.025	82.729 ± 0.028	2.34E−10
			SFA/UFA (%)	16.512 ± 0.130	18.017 ± 0.124	19.218 ± 0.128	1.02E−08
BC_1S_2	84	6	C16:0 (mg/g)	6.723 ± 0.219	7.218 ± 0.181	7.752 ± 0.500	4.86E−02
7784-4Ht × 832			C16:0/ALL (%)	13.098 ± 0.126	14.150 ± 0.115	15.865 ± 0.050	7.42E−04
			SFA/ALL (%)	15.386 ± 0.196	16.390 ± 0.206	18.293 ± 0.501	3.12E−03
			UFA/ALL (%)	83.234 ± 0.068	82.222 ± 0.191	80.247 ± 0.500	1.46E−03
			SFA/UFA (%)	18.511 ± 0.381	19.912 ± 0.357	22.788 ± 0.523	2.59E−03
BC_1S_2	82	7	C16:0 (mg/g)	11.688 ± 0.500	13.128 ± 0.435	14.889 ± 0.577	1.02E−02
By804 × By815			C16:0/ALL (%)	9.407 ± 0.050	11.269 ± 0.055	13.101 ± 0.044	7.16E−07
			SFA/ALL (%)	11.640 ± 0.501	13.464 ± 0.079	15.311 ± 0.048	2.21E−06
			UFA/ALL (%)	87.075 ± 0.500	85.272 ± 0.105	83.425 ± 0.053	5.72E−06
			SFA/UFA (%)	13.401 ± 0.521	15.892 ± 0.101	18.429 ± 0.126	2.57E−06

N and Observed N are the number of genotyped kernels and the number of bulked samples used to determine fatty acids content and composition, respectively. –/–, +/– and +/+ is the homozygous allele of B73, allele that are heterozygous for B73 and By804 and homozygous allele of By804 based on the 11-bp InDel, respectively. The abbreviations of traits can be found in Table S2.
