# Supplementary material for: An 11-bp Insertion in Zea mays fatb Reduces the Palmitic Acid Content of Fatty Acids in Maize Grain
Source: PLoS One. 2011 Sep 13;6(9):e24699. doi: 10.1371/journal.pone.0024699 (PMC3172307; doi:10.1371/journal.pone.0024699)
Supplement: Table S9 — Information for elite inbred lines selected for the gene effect validation of Zmfatb . (PDF) [file pone.0024699.s018.pdf]

Table S9. Information for elite inbred lines selected for the gene effect validation of *Zmfatb*.

Inbred lines	Pedigree	Genotype	C16:0 (mg/g)	C16:0/ALL (%)
By815	BHO	–/–	11.12	9.64
By813	BHO	–/–	11.23	9.17
By804	BHO	+/+	16.47	13.23
By4839	BHO	+/+	16.61	14.56
4F1	Lancaster	–/–	4.91	13.81
Mo17	Lancaster	–/–	5.26	13.85
Ji63	Lancaster	+/+	7.60	18.05
Ji53	Lancaster	+/+	7.61	15.28
Shen5003	Reid	–/–	4.67	14.45
B73	Reid	–/–	5.07	13.89
832	Reid	+/+	7.11	14.76
U8112	Reid	+/+	7.78	15.47
K10	RHO	–/–	4.49	12.98
7884-4Ht	RHO	–/–	5.06	12.65
Ry697	RHO	+/+	12.58	14.97
Sy1035	RHO	+/+	12.74	13.59

–/– and +/+ is the homozygous allele of B73 and By804 based on the 11-bp InDel, respectively; BHO is the Beijing high-oil lines; Lancaster and Reid is the Lancaster and Reid heterotic group, respectively; RHO represents the high-oil population from Reid heterotic group.
